# MicroRNA-382 Promotes M2-Like Macrophage *via* the SIRP-α/STAT3 Signaling Pathway in Aristolochic Acid-Induced Renal Fibrosis

**DOI:** 10.3389/fimmu.2022.864984

**Published:** 2022-05-02

**Authors:** Xiaoyan Wang, Ping Jia, Ting Ren, Zhouping Zou, Sujuan Xu, Yunlu Zhang, Yiqin Shi, Siyu Bao, Yingxiang Li, Yi Fang, Xiaoqiang Ding

**Affiliations:** ^1^ Department of Nephrology, Zhongshan Hospital, Fudan University, Shanghai, China; ^2^ Shanghai Medical Center of Kidney Disease, Shanghai, China; ^3^ Shanghai Institute of Kidney and Dialysis, Shanghai, China; ^4^ Shanghai Key Laboratory of Kidney and Blood Purification, Shanghai, China

**Keywords:** aristolochic acid nephropathy, renal interstitial fibrosis, miR-382, M2 macrophages, SIRP-α, STAT3

## Abstract

Aristolochic acid nephropathy (AAN) is a type of drug-induced nephropathy and is correlated with a potentially progression of kidney fibrosis. However, whether miR-382 is implicated in macrophage activation in AA-induced kidney fibrosis remains elusive. Here, cell-sorting experiments defined a significant miR-382 enrichment in renal macrophage after AAN 14 days. Then, we found that treatment of AA induced a significant switch in the phenotype of macrophage both *in vivo* and *in vitro*. Furthermore, miR-382 knockout (KO) mice and miR-382^-/-^ bone marrow-derived macrophage (BMDM) were subjected to AA induction. We found that both systemic KO and macrophage-specific miR-382 depletion notably suppressed M2-like macrophage activation as well as kidney interstitial fibrosis. Additionally, adoptive transfer of miR-382 overexpression BMDMs into mice promoted AA-induced kidney injury. Moreover, in cultured macrophage, upregulation of miR-382 promoted M2-related gene expression, accompanied by downregulation of signal regulatory protein α (SIRP-α) and activation of signal transducer and activator of transcription 3 (STAT3). The interaction between miR-382 and SIRP-α was evaluated *via* dual-luciferase assay. Knockdown of SIRP-α upregulated phosphorylated STAT3 at S727 and Y705. Pharmacological inhibition of STAT3 was performed both *in vivo* and *in vitro*. Inhibition of STAT3 attenuated AA-induced kidney fibrosis, in parallel to lesser macrophage M2 polarization. Coculture experiments further confirmed that overexpressed miR-382 in macrophage promoted injuries of tubular cells. Luminex bio-chip detection suggested that IL-4 and CCL-5 were critical in the cross talk between macrophages and tubular cells. Taken together, our data suggest that miR-382 is a critical mediator in M2-like macrophage polarization and can be a promising therapeutic target for kidney fibrosis.

## 1 Introduction

Chronic kidney disease (CKD) is a common condition, affecting 10% of all adults globally, with a considerable proportion of patients evolving to end-stage kidney failure, which requires lifelong dialysis or kidney transplantation (1). Several insults contribute to CKD progression, including infections, xenobiotics, toxins, mechanical obstruction, immune complex deposition, chronic infections, and genetic disorders (2). Traditional Chinese medicine has been usually used to treat diseases in Asia for thousands of years. However, Chinese herb-induced nephrotoxicity has been recognized gradually. Aristolochic acid (AA) exists in many kinds of Chinese herbs that causes drug-associated renal injury, as first reported in Belgian patients who had taken AA as part of a slimming regimen (3, 4). Although herbs that are known to contain AAs are no longer permitted in many countries, the incidence of AAN is probably much higher than initially thought, particularly in Asia (5). The progression of AA-induced lesions and mutation is irreversible, and no effective therapeutic treatments for AAN have been established. Activated monocyte/macrophage infiltration is a hallmark of naturally occurring human CKD, as well as of CKD experimentally induced by AA (6).

Tissue macrophages are a major innate immune cell population, characterized by their heterogeneity and plasticity. They are well known for their phagocytic function and are involved in the modulation of homeostasis (7). Traditionally, M1 macrophages are termed as classically activated (activated by interferon-γ) and pro-inflammatory, whereas M2 macrophages are regarded to be alternatively activated (by interleukin-4/interleukin-13) and anti-inflammatory, implicated in repair/regeneration as well as fibrosis. Several studies have confirmed that M2 macrophage accumulation promotes kidney fibrosis through the excessive production of the extracellular matrix and the secretion of profibrotic factors (8–10). However, the mediators of M2 polarization of macrophages as well as their contribution to kidney fibrosis are still unclear.

miR-382, an endogenous, small non-coding RNA, located on chromosome 14q32.31 (11), is critical in various diseases. Considerable studies have reported that miR-382 is a tumor suppressor by regulation of apoptosis or epithelial–mesenchymal transition (EMT) (12, 13). Interestingly, it was reported that more than half of upregulated microRNAs (including miR-382) were localized to the 14q32 microRNA cluster in idiopathic pulmonary fibrosis (IPF) (14), which indicated that the 14q32 microRNA cluster would be critical in organ fibrosis. Moreover, there was nearly a three times upregulation of miR-382 in the human proximal tubular epithelial cell line after induction by transforming growth factor β1 (TGFβ1) (15). Several animal experiments have found the role of miR-382 in renal fibrosis (11, 16–18). However, the specific role of miR-382 in kidney fibrosis still remains unclear. Accordingly, miR-382 may suppress M1 macrophage polarization in bronchopulmonary dysplasia (19). Therefore, it is hypothesized that miR-382 would promote M2 macrophage and contribute to the progression of CKD.

Thus, in this study, we aimed to assess the effects of macrophage miR-382 on M2 polarization and, subsequently, AAN-associated interstitial fibrosis and elucidate the underlying mechanisms.

## 2 Materials and Methods

### 2.1 Animal Studies

The male adult mice (8 to 10 weeks old; 20–25 g) used in these experiments were housed in specific-pathogen-free conditions and allocated to age- and sex-matched groups. The mice were obtained from SLAC Laboratory Animal Co., Ltd., Shanghai, China.

#### 2.1.1 Ethics

All animal experiments were approved by the Institutional Animal Care and Use Committee of Fudan University and were performed in accordance with the National Institutes of Health Guide for the Care and Use of Laboratory Animals. The experimental unit was the individual animal, each independently allocated to a treatment group.

#### 2.1.2 microRNA-382 Knockout Mice

miR-382^-/-^ mice (back-crossed to C57BL/6 mice for 10 generations) were established by Bioray Laboratories (Shanghai, China). The identification of miR-382^-/-^ mice is shown in **Figure S11A**. The primer sequence used for the identification of miR-382 knockout mice is displayed in **Supplementary Table 2**.

#### 2.1.3 Macrophage-Specific miR-382 Depletion Mice

MiR-382^flox/flox^ mice on a C57BL/6J background were established by Cyagen Laboratories (Santa Clara, CA, USA). Lyz2-Cre mice were purchased from the Shanghai Model Organisms Center (Shanghai, China). MiR-382^flox/flox^ Lyz2-Cre mice were generated using the Cre-loxP system by crossing miR-382^flox/flox^ with Lyz2-Cre mice. The identification of miR-382^flox/flox^ Lyz2 cre^+^ mice is shown in **Figure S3A**. The primer sequences used for the identification of miR-382^flox/flox^ and Lyz cre^+^ mice are shown in **Supplementary Table 2**.

#### 2.1.4 Aristolochic Acid Nephropathy Model

A chronic AA model was induced by injection of AA (10 mg/kg, i.p.; A9451, Sigma-Aldrich, St. Louis, MO) for 1, 3, 7, 14, and 28 days. miR-382^-/-^ mice were injected with AA for 7, 14, and 28 days. MiR-382^flox/flox^ Lyz2-Cre^+^ or miR-382^flox/flox^ Lyz2-Cre^-^ mice were treated with AA for 14 days. Control mice were treated with the same dosage of saline *via* intraperitoneal injection. Kidney tissue and blood samples were obtained for further analysis.

#### 2.1.5 Pharmacological Inhibition of STAT3

Inhibition of STAT3 *in vivo* was performed. STA-21 (HY-18061, MedChemExpress, Princeton, NJ, USA) was administered intraperitoneally to animals at a dose of 0.5 mg/kg the day before AA injection (DMSO was used as control). The same dosage of STA-21 was repeated once daily for 2 weeks.

#### 2.1.6 Depletion of Macrophages

Macrophage depletion in mice was performed. Mice were divided into 4 groups: normal group, AA group, LPBS+AA group, and LC+AA group. Liposomal clodronate or the same volume of control liposome suspension (catalog no. CLD-8938, Encapsula Nanosciences LLC, Nashville, TN) was administered intraperitoneally to the animals at a dose of 200 μl on the day before AA injection, and the treatment was repeated every 4 days (20). Animals were sacrificed after 14 days. Kidney tissues and blood samples were obtained for further analysis.

#### 2.1.7 Adoptive Transfer Macrophages

Bone marrow-derived macrophages (BMDMs) were isolated from mice and induced in the presence of 30% L929 supernatant (21). After 7 days of induction, miR-382 mimic or NC were transfected into BMDMs. As previously reported, macrophages were depleted and reconstituted (22). Firstly, to deplete macrophages, LC was administered intraperitoneally at a dose of 200 μl in mice. Subsequently, AA was administrated *via* intraperitoneal injection at a dosage of 8 mg/kg. Then, overexpression of miR-382 BMDMs or NC BMDMs was counted and resuspended to a concentration of 10^7^/ml with PBS. 100 μl (10^6^ BMDMs) was slowly injected *via* the tail vein and repeated every four days. After 14 days of AA treatment, kidney tissues and blood samples were obtained for further analysis.

#### 2.1.8 Kidney Macrophage Isolation by Flow Cytometry

Kidney macrophage isolation at day 14 of AAN was performed *via* kidney digestion and incubation with Fc Block (catalog No. 14-0161-85, eBioscience, San Diego, CA, USA). Cells were incubated with anti-CD45-APC/Cyanine7 (catalog no. 103115, BioLegend, San Diego, CA, USA), anti-CD11b-FITC (553310, BD, Franklin Lakes, NJ, USA), and anti-F4/80-PE (12-4801-80, eBioscience). CD45^+^ cells were first selected, and CD11b^+^F4/80^+^ cells were then gated for isolation *via* flow cytometry (BD FACSAria II). Total RNA was extracted from CD45^+^ CD11b^+^F4/80^+^ cells and reverse-transcribed *via* RT-qPCR analysis, as described below (23).

#### 2.1.9 Flow Cytometry Analysis of Kidney Tissues

Kidney single-cell suspensions were prepared *via* mechanical and enzymatic digestion as previously described (23). Suspensions were incubated with Fc Block anti-mouse CD16/32 (catalog no. 14-0161-85, eBioscience) for 15 min and then treated with anti-CD45-APC/Cyanine7 (catalog no. 103115, BioLegend), anti-CD11b-FITC (553310, BD), anti-F4/80-PE (12-4801-80, eBioscience), anti-CD206-Alexa Fluor 647 (565250, BD), and anti-CD86-eFluor 450 (48-0862-82; eBioscience) for 30 min at 4°C. Cells were washed with PBS, resuspended with 200 μl PBS, and detected using a Focusing Cytometer (Thermo Fisher Scientific, Waltham, MA, USA). Data were analyzed using FlowJo software version 10.

#### 2.1.10 Assessment of Serum Creatinine

Serum creatinine was determined in 30 μl of serum using the QuantiChrom™ Creatinine Assay Kit (DICT-500, BioAssay Systems, Hayward, CA, USA).

#### 2.1.11 Histological Analysis and Immunohistochemistry Staining

Renal tissues were fixed with 10% formalin, embedded in paraffin wax, and sliced into 4-μm-thick sections for HE staining, Masson staining, Sirius Red staining, or immuno-histochemical staining. Immunohistochemical staining was performed as previously described (11). Primary antibodies used included anti-α-SMA (ab5694, Abcam, Cambridge, MA, USA), anti-Collagen I (ab34710, Abcam), anti-Fibronectin (F3648, Sigma, St. Louis, MO, USA), anti-Vimentin (ab137321, Abcam), anti-p-STAT3 S727 (ab32143, Abcam), anti-p-STAT3 Y705 (9145S, CST, Danvers, CA, USA), anti-STAT3 (9139T, CST), anti-CD206 (ab64693, Abcam), anti-Arg-1 (ab233548, Abcam), and anti-Ym1 (ab192029, Abcam). The secondary antibody used was HRP-conjugated goat anti-rabbit IgG Abs (#ZB-2301, Zhongshan Gold Bridge Biotechnology, China). Sections were evaluated *via* microscopy (×200 magnification, Leica DM 6000B; Leica Microsystems, Wetzlar, Germany). Histological analysis was assessed in a blinded manner.

#### 2.1.12 Immunofluorescence of Kidney Sections

Frozen kidney tissues were embedded in OCT and sliced into 6-μm-thick sections for immunofluorescence. Sections were fixed in 4% PFA for 15 min and then permeabilized using 0.5% Triton X-100 in PBS for 10 min. After blocking with 5% BSA in PBS for 1 h at room temperature, they were incubated with antibodies, anti-F4/80 (70076, CST), anti-CD206 (ab64693, Abcam), and anti-p-STAT3 Y705 (ab191419, Abcam) overnight at 4°C. The secondary antibodies used included Donkey anti-Rabbit IgG-AlexaFluor 488 (abs20020, Absin), Donkey anti-Rabbit IgG-Cy3 (abs20022, Absin), and Donkey anti-Mouse IgG-AlexaFluor 594 (abs20017, Absin). The nuclei were stained with Hoechst33258. Images were acquired using an Olympus FV3000 confocal microscope.

### 2.2 *In Vitro* Studies

#### 2.2.1 Bone Marrow-Derived Macrophages Isolation

BMDMs were isolated and cultured in the presence of L929 supernatant as previously described (21). L929 cells were a gift from the Urology Department of Zhongshan Hospital affiliated with Fudan University. Briefly, L929 cells were cultured with RPMI 1640 (catalog no. 31870082, Gibco, Grand Island, NY, USA), 10% FBS (catalog no. 10100147, Gibco), and 1% penicillin–streptomycin (catalog No. 15140163, Thermo Fisher) for 3 days, and supernatant was collected. Bone marrow cells were obtained from WT mice or miR-382^-/-^ mice, and their differentiation was induced using RPMI 1640, 30% L929 supernatant, and 10% FBS for 7 days. The medium was then changed to complete medium without L929 supernatant. For *in vitro* treatment, BMDMs were stimulated with 10 μg/ml AA for 0, 6, 12, 24, and 48 h. Alternatively activated macrophages were induced using 50 ng/ml murine recombinant IL-4 (404-ML-010, R&D, Minneapolis, MN, USA) for 48 h.

#### 2.2.2 Raw264.7 and Mouse Tubular Epithelial Cell Culture

Raw264.7 cells were purchased from the American Type Culture Collection (ATCC) and mouse renal tubular epithelial cells (MTECs) from Caltag Medsystems (Buckingham, UK) and cultured in DMEM high glucose (catalog no. 11965092, Gibco), 10% FBS, and 1% penicillin–streptomycin. For *in vitro* treatment, Raw264.7 cells were stimulated with 10 μg/ml AA for 0, 6, 12, 24, and 48 h.

#### 2.2.3 Cell Transfection or Intervention

The transgene expression of miR-382 was induced with 100 nM anti-miR-382 (also referred to as anti-scramble) or miR-382 mimics (also referred to as negative control) (Exiqon, Shanghai, China) for 24–36 h. Inhibition of SIRP-α was performed by transfecting cells with SIRP-α siRNA or a negative control (100 nM, sc-36493, Santa Cruz Biotechnology). Inhibition of STAT3 was performed using STA-21 (20 μM, HY-18061, MedChemExpress) or STAT3 siRNA for 12 h prior to AA treatment. Overexpression of STAT3 was established using a murine STAT3 plasmid (Zorin Biotechnology Co. Ltd., Shanghai, China) transfected for 48 h.

#### 2.2.4 Coculture Experiments

The effects of macrophages on MTECs were evaluated using a coculture system as previously described (24). Briefly, Raw264.7 cells were subjected to AA treatment for 24 h or anti-miR-382/anti-scramble (or miR-382 mimics/NC) for 12 h followed by AA treatment. The medium was then changed to conditional medium (CM) for 24 h. CM was collected, and MTECs were incubated for 36 h. Total protein was extracted from MTECs and subjected to Western blot analysis.

#### 2.2.5 Luminex Liquid Suspension Chip Detection

Luminex liquid suspension chip detection was performed by Wayne Biotechnologies (Shanghai, China). The Bio-Plex Pro Mouse Cytokine Grp I Panel 23-plex was applied according to the manufacturer’s instructions. Briefly, conditional medium (50 μl) of Raw264.7 cells from control, AA, anti-scramble+ AA, or anti-miR-382+ AA groups was incubated in 96-well plates embedded with microbeads for 30 min and then incubated with a detection antibody for 30 min. Subsequently, streptavidin-PE was added into 96-well plates for 10 min and values were read by the Bio-Plex MAGPIX System (Bio-Rad).

#### 2.2.6 Flow Cytometry of Cultured Macrophages

The polarization of macrophages *in vitro* was detected *via* flow cytometry. Briefly, Raw264.7 cells and BMDMs were collected from 6-well plates after AA stimulation and washed with PBS three times. The cell suspension was collected into 1.5-ml EP tubes and incubated with Fc Block anti-mouse CD16/32 on ice for 15 min. After washing twice, cells were incubated with anti-CD11b-FITC, anti-F4/80-PE, anti-CD206-Alexa Fluor 647, and anti-CD86-eFluor 450 on ice for 30 min in the dark. After washing twice, cells were suspended in 200 μl with 1% FBS and then detected using an Acoustic Focusing Cytometer (Thermo Fisher Scientific). Data were analyzed using FlowJo software version 10.

#### 2.2.7 Immunofluorescence of Cultured Macrophages

Raw264.7 cells were mounted on slides, and treatments were performed. After treatment, slides were fixed in 4% PFA for 15 min and then permeabilized using 0.5% Triton X-100 in PBS for 10 min. After blocking with 5% BSA in PBS for 1 h at room temperature, they were incubated with antibodies, including anti-CD206 (ab64693, Abcam), anti-Arg-1 (ab233548, Abcam), anti-Ym1 (ab192029, Abcam), anti-SIRPα (ab191419, Abcam), and anti-p-STAT3 S727(ab32143, Abcam) overnight at 4°C. The secondary antibodies were as described above. The nuclei were stained with Hoechst33258. Images were acquired using an Olympus FV3000 confocal microscope.

#### 2.2.8 Dual-Luciferase Assay

The TargetScan bioinformatics website (http://www.targetscan.org) was utilized to predict targets of miR-382 and the possible sequences of miR-382 target binding sites. pMIR-SIRPa-3′ UTR-wt and pMIR-SIRPa-3′ UTR-mut were cloned into the pMiR dual-luciferase reporter plasmid vector. The recombinant pMiR dual-luciferase reporter plasmid was co-transfected with miR-382 mimics or a negative control into Raw264.7 cells using Lipo3000 Reagent. Dual-luciferase activity was measured using the Dual-Glo Luciferase Assay System.

### 2.3 Western Blot Analysis

Western blot was performed as previously described (11). The primary antibodies used included anti-CD206 (ab64693, Abcam), anti-Arg-1 (ab233548, Abcam), anti-Ym1 (ab192029, Abcam), anti-SIRPα (ab191419, Abcam), anti-p-STAT3 S727 (ab32143, Abcam), anti-p-STAT3 Y705 (#9664, CST), anti-STAT3 (9662, CST), anti-α-SMA (ab7817, Abcam), anti-cleaved Caspase 3 (CST; 9662), anti-Caspase 3 (CST; #3498), anti-Bcl2 (CST; #3498), anti-E-cadherin (GTX100443, GTX), and anti-GAPDH (ab181602, Abcam). The secondary antibodies included Peroxidase AffiniPure Goat Anti-Rabbit IgG (H+L) and Peroxidase AffiniPure Goat Anti-Mouse IgG (H+L) (111035003, 115035003, Jackson, West Grove, PA, USA). Protein levels were quantified using the Image Lab software, version 3.0 (Bio-Rad, Hercules, CA, USA).

### 2.4 Real-Time RT-qPCR

Total RNA from Raw264.7, BMDMs, and kidney tissues were extracted using TRIzol and reverse-transcribed into cDNA using the PrimeScript™ RT Reagent Kit. The 18S rRNA gene was used to normalize gene expression, including Fizz1, Arg-1, Ym-1, IL-10 (interleukin-10), IL-6 (interleukin-6), TNF-α (tumor necrosis factor-α), iNOS, Col1, and TGF-β1. The expression of miR-382 was reverse-transcribed by MultiScribe™ Reverse Transcriptase Kit (4311235, Applied Biosystems, Foster City, CA, USA) and detected using TaqMan probes (000572, Life Technologies, Carlsbad, CA, USA). miR-382 expression was normalized to that of U6. PCR primer sequences are shown in **Supplementary Table 1**.

### 2.5 Statistical Analysis

All experiments were performed in biological replicates. All the experiments were replicated at least twice. All *in vivo* and *in vitro* experiment samples were randomized. Data were analyzed using GraphPad Prism Software and expressed as the mean ± standard error of the mean. Two-tailed, unpaired Student’s tests were performed to determine the significance of differences between two groups. ANOVA was used to analyze intergroup differences. Statistical significance was set at *P* < 0.05.

### 2.6 Role of the Funding Source

This work was supported by the Science and Technology Commission of Shanghai (14DZ2260200) and the National Natural Science Foundation of China grants 91849123 (to XD) and 81870466 (to PJ).

## 3 Results

### 3.1 Phenotype Switch of Macrophage Occurs in AA Nephropathy

**Figure 1:**
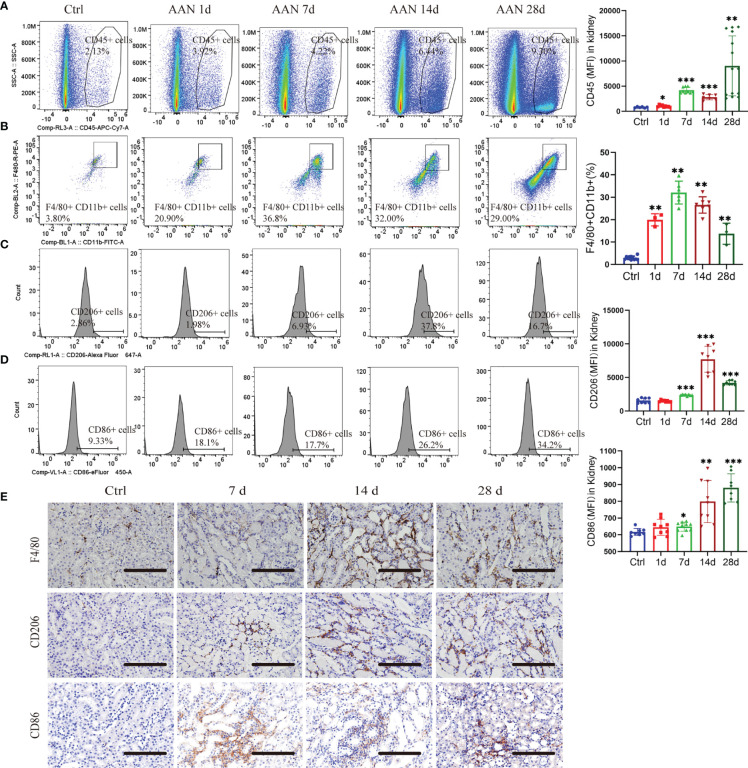
Macrophages undergo polarization during AA nephropathy. **(A)** Representative flow cytometry plots and mean fluorescence intensity (MFI) of CD45+ leukocytes in renal in control and 1, 3, 7, 14, and 28 days of AAN. **(B)** Representative flow cytometry plots and percentage of CD11b+F4/80+ macrophages in kidney in control and 1, 3, 7, 14, and 28 days of AAN. **(C)** Representative flow cytometry images and MFI of CD206+ macrophages in control and 1, 3, 7, 14, and 28 days of AAN. **(D)** Representative flow cytometry images and MFI of CD86+ macrophages in control and 1, 3, 7, 14, and 28 days of AAN. **(E)** Representative images of immunostaining for F4/80 and CD206 in renal sections in control and 7, 14, and 28 days of AAN. Scale bars, 200 μm. n = 6 mice each group. *P < 0.05; **P < 0.01; ***P < 0.001 ANOVA.

In CKD, persistent M2 macrophage infiltration may promote kidney fibrosis (25). To study the role of the macrophages of kidneys in AAN, we determined their number and polarization *via* flow cytometry. Leukocytes were distinguished as CD45^+^, and macrophages were further analyzed as CD11b^+^/F4/80^+^. CD206^+^ macrophages were regarded as M2 and CD86^+^ as M1 macrophages (26). The number of CD45^+^ leukocytes increased during AAN (**Figure 1A**). A robust increase in CD11b^+^/F4/80^+^ macrophages through the course of AAN (**Figure 1B**). Interestingly, both CD86^+^ M1 and CD206^+^ M2 macrophages increased notably on days 7, 14, and 28 after AA injection (**Figures 1C, D**). Consistently, immunohistochemistry for anti-F4/80, anti-CD206, and anti-CD86 in renal sections showed the accumulation of macrophages and M2-like macrophages and M1-like macrophages in AAN (**Figure 1E**). Thus, our data showed significant activation of macrophage in kidney in the progression of AAN.

### 3.2 Depletion of Macrophages Reduces AA-Induced Renal Fibrosis

To explore the function of renal macrophages in AAN, liposomal clodronate (LC), which was previously reported to inhibit macrophage influx (27), was administered in AAN mice at 14 days (PBS liposomes were used as control treatment). *Via* flow cytometry, we confirmed that LC injection efficiently depleted renal macrophages (F4/80^+^/CD11b^+^ macrophages) (**Figure S1A**). Immunofluorescence for F4/80^+^ macrophages in renal tissue further confirmed the efficiency of LC (**Figure S1B**). Further, α-SMA protein levels in renal tissue were significantly downregulated after LC treatment (**Figure S1C**). Macrophage depletion suppressed the mRNA expression of TGF-β1 and collagen I (**Figure S1D**). Taken together, the current results suggest a central role of renal macrophages in the progression of AA-induced kidney fibrosis.

### 3.3 MicroRNA-382 Correlates With Macrophage M2 Polarization and Renal Fibrosis in an AAN Murine Model

**Figure 2:**
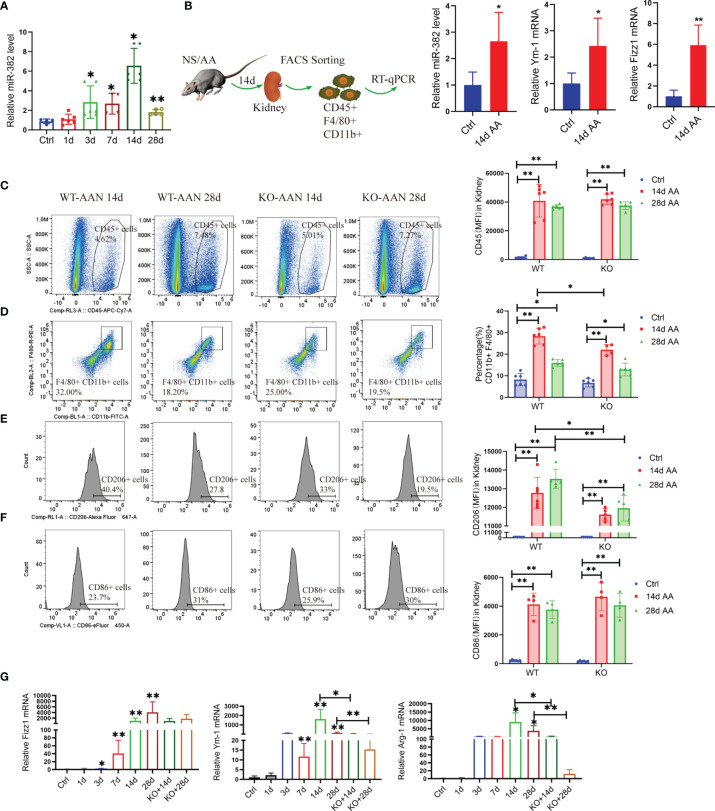
Blockade of miR-382 inhibits M2-like macrophage polarization in the kidney. **(A)** Relative abundance of miR-382 after intraperitoneal injection of 10 mg/kg AA at a concentration at 0.5 mg/ml for 1, 3, 7, 14, and 28 days; saline was administered as control treatment. U6 was used as the endogenous control; n = 6 per group. **(B)** Macrophages were identified as CD45+CD11b+F4/80+ by flow cytometry and isolated from the kidneys from normal and 14 days of AAN. Real-time RT-qPCR was performed in isolated macrophages. Transcripts of miR-382, Fizz1, and Ym-1 of sorting macrophages from normal and fibrotic kidneys (14 days AAN). U6 was used an endogenous control of miR-382, and 18S serves as standard of Fizz1 and Ym-1; n = 3 mice each group. **(C)** Representative flow cytometry plots of CD45+ leukocytes in renal between WT and KO mouse after 14 and 28 days of AAN. Mean fluorescence intensity (MFI) of CD45 among ctrl and AAN 14 days and AAN 28 days from WT or KO mice. **(D)** Representative flow cytometry plots of CD11b+F4/80+ macrophages in kidney between WT and KO mouse after 14 and 28 days AAN. Percentage of CD11b+F4/80+ macrophages among ctrl and AAN 14 days and AAN 28 days from WT or KO mice. **(E)** Representative flow cytometry images and MFI of CD206+ macrophages between WT and KO mouse after 14 and 28 days AAN. Mean fluorescence intensity (MFI) of CD206 among ctrl and AAN 14 days and AAN 28 days from WT or KO mice. **(F)** Representative flow cytometry images and MFI of CD86+ macrophages between WT and KO mouse after 14 and 28 days AAN. Mean fluorescence intensity (MFI) of CD86 among ctrl and AAN 14 days and AAN 28 days from WT or KO mice. **(G)** Relative mRNA levels of Fizz1, Arg-1, and Ym-1 in renal tissue from control mice, AAN 1, 3, 7, 14, and 28 days mice as well as AAN 14 and 28 days KO mice. 18S was used as an endogenous control; n = 6 mice per group. *P < 0.05; **P < 0.01; ANOVA.

To explore the role of miR-382 in the promotion of macrophage M2 polarization and AA-induced CKD, we subjected miR-382 knockout (KO) mice and macrophage-specific depletion miR-382 mice (MKO) to AA-induced nephropathy. We first performed a time-course study using mouse models of AAN (1, 3, 7, 14, and 28 days; NS as control). In AA-treated mice, renal miR-382 levels gradually increased throughout the experiment and peaked at 14 days of AAN (**Figure 2A**). Furthermore, we isolated CD45+CD11b+ F4/80+ cells *via* flow cytometry and found a large induction of miR-382 expression as well as Fizz1 and Ym-1 mRNA levels on macrophages from the fibrotic kidneys, compared with normal kidneys (**Figure 2B**), supporting the hypothesis of involvement of miR-382 in the regulation of M2 polarization.

**Figure 3:**
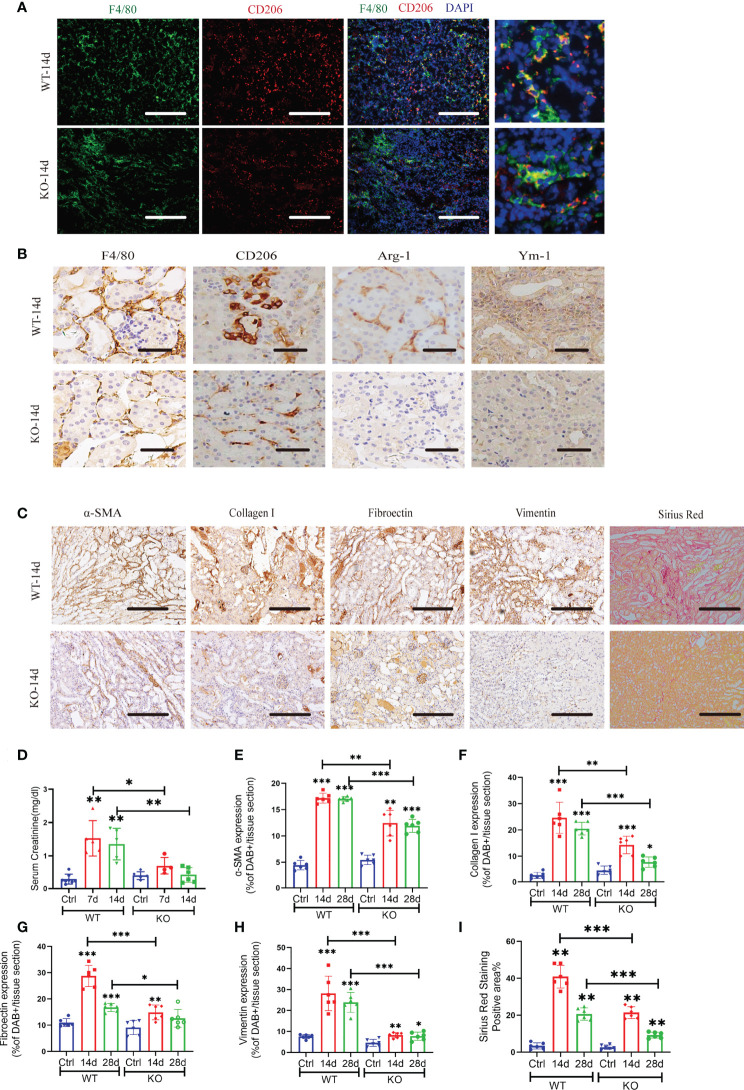
Blockade of miR-382 inhibits M2 polarization in the kidney and attenuates AA-induced CKD. **(A)** Images of co-staining for F4/80, CD206, and DAPI in renal sections from WT-14 days and KO-14 days. F4/80 was marked as green. CD206 was marked as red. Nucleic was blue. Scale bars, 200 μm. **(B)** Representative immunostaining for F4/80, CD206, Arg-1, and Ym-1 in WT and KO mice of 14 days of AAN. Scale bars, 20 μm. **(C)** Representative Sirius Red staining and immuno-histochemistry staining with antibodies against α-SMA, collagen I, fibronectin, and vimentin in renal sections from WT and KO of 14d AAN. Scale bars, 200 μm. **(D)** Serum creatinine of wild-type (WT) and miR-382 knockout (KO) mice with AA treatment for 7 and 14 days; saline was administered as control treatment. **(E–H)** Quantification of positive area for α-SMA, collagen I, fibronectin, and vimentin. Five microscopical fields were randomly selected per section, and the average positive area was calculated; n = 6 mice per group. **(I)** Quantification of mean positive area for Sirius Red staining. Five microscopical fields were randomly selected per section, and the average positive area was calculated; n = 6 mice per group. *P < 0.05; **P < 0.01; ***P < 0.001; ANOVA.

We analyzed the injured kidney from WT and KO mice for 14 and 28 days of AAN *via* flow cytometry. As the results show, miR-382 deficiency did not affect the number of CD45^+^ leukocytes and CD86+ macrophages but decreased CD11b+F4/80 macrophages at 14 days of AAN (**Figures 2C, D, F**). Of note, ablation of miR-382 significantly suppressed CD206^+^ macrophages in the kidney at both 14 and 28 days of AAN (**Figure 2E**). In addition, we analyzed normal kidneys from WT and KO mice and we found no differences between them on CD45+, CD11b+F4/80+, CD86+, and CD206+ cells. Representative flow cytometry images are shown in **Figure S2A**, and quantification of flow cytometry data is shown in **Figures 2C–F**. AA injection could largely increase Fizz1, Arg-1, and Ym-1 expression in renal as early as 3 days and up until 28 days, but it was inhibited in KO mice at 14 and 28 days of AAN (**Figure 2G**), suggesting that miR-382 would promote renal M2-like macrophage polarization in AAN. Co-staining for F480 and CD206 in the kidney further demonstrated that the percentage of CD206-positive macrophages was inhibited notably in miR-382 KO mice after 14 days of AAN (**Figure 3A**). Immunohistochemistry for F4/80, CD206, Arg-1, and Ym-1 in renal sections indicated that alternatively activated macrophages were significantly suppressed in miR-382 KO kidneys (**Figure 3B**).

Moreover, miR-382 could also regulate AA-induced CKD. Serum creatinine of mice increased significantly at 7 and 14 days after AA injection, while depletion of miR-382 alleviated renal dysfunction (**Figure 3D**). Sirius Red staining revealed significantly enhanced collagen deposition in WT kidneys relative to KO kidneys following AA treatment (**Figures 3C, I**). Immunohistochemical staining showed a decline in α-SMA, fibronectin, and vimentin and collagen I expression in KO kidneys, compared with WT kidneys (**Figures 3C, E–H**).

Additionally, we generated mice with macrophage-specific miR-382 depletion (miR-382^flox/flox^ Lyz2-Cre+, or “MKO”) and wild-type littermate control subjects (miR-382^flox/flox^ Lyz2-Cre-, or “CKO”) and subjected these animals to the AAN model for 14 days. There were no differences between CKO and MKO mice in CD45+ leukocytes, CD11b+F4/80+ macrophages, and CD86+ M1-like macrophages (**Figure S3B-D**). Of note, consistent with our findings on KO mice, MFI of CD206+ M2-like macrophages in renal was inhibited notably in MKO mice, compared with CKO mice (**Figure S3E**). Compared to the CKO mice, the protein expression of α-SMA after AA induction was significantly inhibited in renal, indicating that depletion of miR-382 on macrophages would be reno-protective (**Figure S3F**). AA-induced tubule atrophy, deposition of collagen, or expression of α-SMA, collagen I, and fibronectin was relieved remarkably in MKO mice, compared with CKO mice (**Figure S3G**). Collectively, our data showed that renal macrophage plays a critical role in AA-induced kidney fibrosis. Ablation of miR-382 could inhibit renal macrophage M2 polarization and AA-induced CKD. Thus, it is our hypothesis that miR-382 would regulate AA-induced CKD by promoting M2-lile macrophage polarization.

### 3.4 Adoptive Transfer miR-382 OE BMDMs Augments AA-Induced CKD

**Figure 4:**
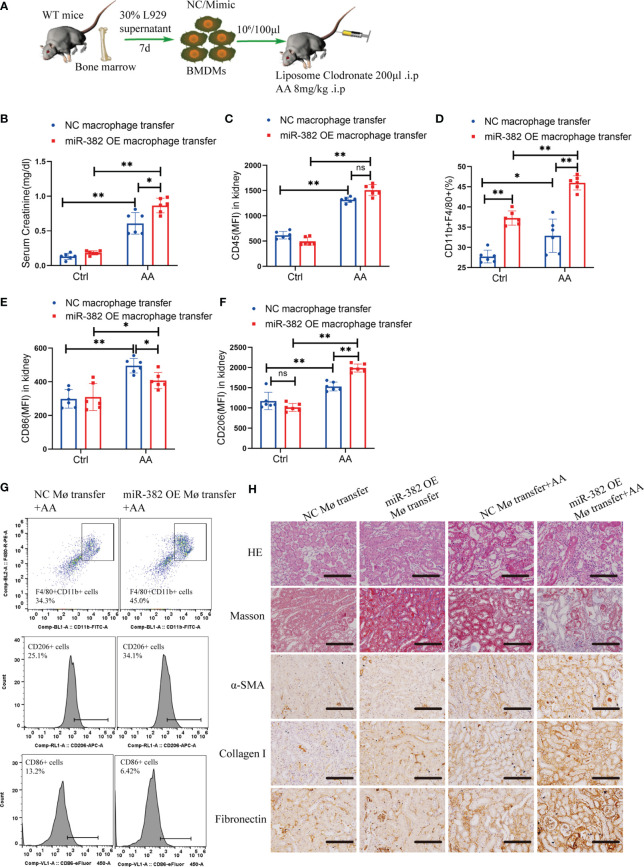
MiR-382 OE BMDMs transfer augmented AA-induced CKD. **(A)** Strategy of macrophages transfer in mice: bone marrow was acquired from mice and further induced with 30% L929 supernatant and derived into BMDMs. NC or miR-382 mimic were separately transfected into BMDMs. Macrophages were depleted with LC via intraperitoneally injection (200μl) in mice. AA was administrated at a dosage of 8mg/kg in mice. Then, BMDMs were counted and suspended in PBS (107/ml) and injected with 100μl via tail vein and repeated every four days. **(B)** Serum creatinine of mice between NC macrophage transfer and miR-382 OE macrophage transfer groups. **(C-F)** MFI of CD45, percentage of CD11b+F4/80+, MFI of CD86 and MFI of CD206 between NC macrophage transfer and miR-382 OE macrophage transfer groups. **(G)** Representative images of CD11b+F4/80+ macrophages, CD206+ M2 macrophages and CD86+ M1 macrophages between NC macrophage transfer and miR-382 OE macrophage transfer groups. **(H)** Representative images for HE staining, Masson staining, IHC for α-SMA, Collagen I and Fibronectin in renal sections among these groups. Scale bars, 200μm. n=6 mice each group. *P < 0.05; **P < 0.01; ANOVA. ns, no significance.

To further explore the role of miR-382 in macrophages in the AA-induced CKD, we depleted and reconstituted macrophages in mice. Bone marrow-derived macrophages (BMDMs) were transfected miR-382 mimic and then transferred into mice with AA induction (**Figure 4A**). In AA induction, the serum creatinine of miR-382 OE BMDM transfer mice was higher than that of NC BMDM transfer mice (**Figure 4B**). Interestingly, adoptive transfer miR-382 OE BMDMs could raise more CD11b+F4/80+ macrophages in kidney in both control and AA mice but preserved CD45+ leukocytes (**Figures 4C, D**). In addition, adoptive transfer miR-382 OE BMDMs also triggered macrophage M1-to-M2 transition (**Figures 4E, F**). Representative flow cytometry images are displayed in **Figure 4G**. Moreover, adoptive transfer miR-382 OE BMDMs could augment AA-induced renal injury and fibrosis according to HE staining, Masson staining, IHC for α-SMA, collagen I, and fibronectin (**Figure 4H**). These results supported the role of miR-382 in macrophages for promoting the recruitment of macrophages in kidney, macrophage M1-to-M2 transition, and aggravating of AA-induced injuries and fibrosis.

### 3.5 AA Regulates the Phenotype of Cultured Macrophages

**Figure 5:**
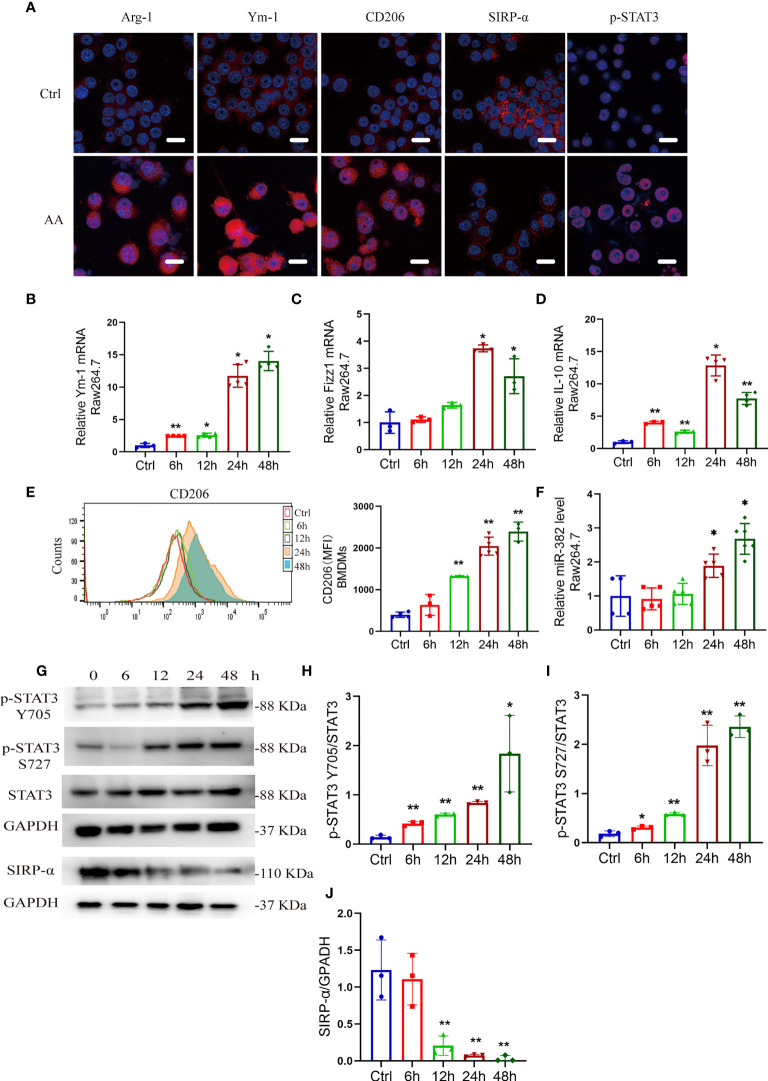
AA induces M2-like macrophage polarization, accompanied by upregulation of miR-382 and phosphorylated STAT3 and downregulation of SIRP-α. **(A)** Immunofluorescence staining for Arg-1, Ym-1, CD206, SIRP-α, and p-STAT3 in Raw264.7 cells with NC or AA (10 μg/ml 48 h) induction. Scale bars, 20 μm. **(B–D)** mRNA levels of Ym-1, Fizz1, and IL-10 in Raw264.7 cells following AA treatment. 18S was used as an endogenous control. **(E)** Flow cytometry of CD206^+^ macrophages among bone marrow-derived macrophages (BMDMs) in the time course of AA treatment. **(F)** Levels of miR-382 in Raw264.7 cells in the time course; saline was administered as control treatment. U6 was used as an endogenous control. **(G)** Images of Western blot for p-STAT3 Y705, p-STAT3 S727, STAT3, and SIRP-α in Raw264.7 after treating AA 6, 12, 24, and 48 h. GAPDH served as standard. n = 3 holes each group. **(H–J)** Quantification of p-STAT3 Y705 and p-STAT3 S727 in the time course of AA treatment in Raw264.7 cells. STAT3 served as standard; quantification of SIRP-α in the time course with AA exposure. GAPDH served as standard. *P < 0.05; **P < 0.01; ANOVA.

To further explore the effect of AA on macrophages, we treated BMDMs and Raw264.7 with AA (10 μg/ml). BMDMs were identified as CD11b^+^/F4/80^+^ *via* flow cytometry (**Figure S4A**). After 48 h of AA induction, the expression of Arg-1, Ym-1, and CD206 increased in Raw264.7 cells, accompanied by loss of SIRP-α and upregulation of p-STAT3, as immunofluorescence staining shown in **Figure 5A**. The mRNA levels of Ym-1, Fizz1, and IL-10 were notably elevated at 24 and 48 h (**Figures 5B–D**). *Via* flow cytometry, the mean fluorescence intensity (MFI) of CD206 was significantly enhanced at 12, 24, and 48 h after AA treatment (**Figure 5E**). Abundance of miR-382 increased significantly as AA induction (**Figure 5F**). In addition, the ratio of p-STAT3 S727/STAT3 and p-STAT3 Y705/STAT3 increased gradually in AA induction but SIRP-α decreased significantly at 12, 24, and 48 h after AA treatments (**Figures 5G–J**).

Of note, we found that both M1 and M2 macrophages coexisted in the kidney of AAN. Interestingly, in an *in vitro* experiment, we also found that the expression of CD86 increased gradually after AA treatment *via* flow cytometry and the mRNA levels of IL-6, TNFα, and iNOS were significantly enhanced as early as 6 h after AA treatment (**Figures S4B, C**), indicating that macrophage M1 polarization was induced in the early stage of AA induction. However, whether miR-382 could affect the phenotype of macrophage remained unknown.

### 3.6 Suppression of miR-382 Hinders Macrophage M2 Polarization *In Vitro*

**Figure 6:**
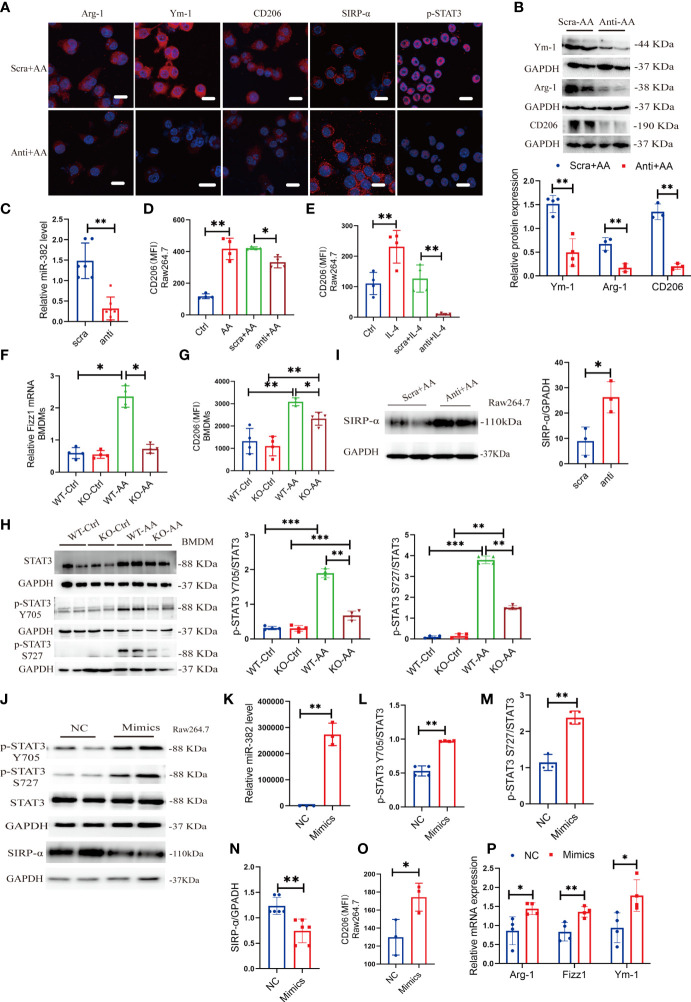
Suppression or overexpression of miR-382 is implicated in M2-like macrophage activation *in vitro.* **(A)** Immunofluorescence staining for Arg-1, Ym-1, CD206, SIRP-α, and p-STAT3 in Raw264.7 cells with anti-scramble or anti-miR-382 with AA (10 μg/ml 48 h) induction. Scale bars, 20 μm. **(B)** Representative Western blot images and relative quantification for Ym-1, Arg-1, and CD206 in Raw264.7 cells between anti-scramble+ AA and anti-miR-382+ AA groups. **(C)** Abundance of miR-382 in anti-scramble or anti-miR-382 with AA (10 μg/ml 48 h) induction. U6 was used as an endogenous control. **(D)** MFI of CD206+ macrophages among control, AA, anti-scramble +AA and anti-miR-382+ AA groups in Raw264.7 cells. **(E)** MFI of CD206+ macrophages among control, IL-4, anti-scramble +IL-4, and anti-miR-382+ IL-4 groups in Raw264.7 cells. **(F)** mRNA level of Fizz1 in BMDMs from WT and KO mice following AA treatment. 18s served as standard. **(G)** MFI of CD206+ macrophages in BMDMs from WT and KO mice following AA treatment. **(H)** Images and quantification of Western blot for p-STAT3 Y705, p-STAT3 S727, and STAT3 in BMDMs between WT and KO mice following AA treatment. GAPDH was used an endogenous control. **(I)** Images and quantification of Western blot for SIRP-α in Raw264.7 cells in anti-scramble and anti-miR-382 with AA treatment. GAPDH served as standard. **(J)** Images of Western blot for p-STAT3 Y705, p-STAT3 S727, STAT3, and SIRP-α in NC and miR-382 mimic groups in Raw264.7 cells. GAPDH served as standard. **(K)** Abundance of miR-382 between NC and Mimics groups in Raw264.7 cells. U6 was used as an endogenous control. **(L, M)** Ratio of p-STAT3 Y705/STAT3 and p-STAT3 S727/STAT3 in NC and miR-382 mimic groups in Raw264.7 cells. **(N)** Quantification of Western blot for SIRP-α in Raw264.7 cells in NC and Mimics groups. **(O)** MFI of CD206+ macrophages between NC and Mimics groups in Raw264.7 cells. **(P)** Relative mRNA levels of Arg-1, Fizz1, and Ym-1 in Raw264.7 cells after overexpression of miR-382. *P < 0.05; **P < 0.01; ***P < 0.001; ANOVA.

To further explore the role of miR-382 on polarization of macrophage, locked nucleic acid (LNA)-modified anti-miR-382 oligo was used to knock down miR-382 and bone marrow-derived macrophages (BMDMs) were acquired from WT and miR-382 KO mice, respectively. According to the immunofluorescence staining and Western blot analysis, expressions of Arg-1, Ym-1, and CD206 were suppressed after miR-382 downregulation in AA induction, compared with the anti-scramble group (**Figures 6A, B**). Abundance of miR-382 declined significantly after anti-miR-382 transfection in Raw264.7 cells (**Figure 6C**). Knockdown of miR-382 within AA treatment could partly inhibit CD206 expression in Raw264.7 cells (**Figure 6D**). IL-4 treatment is a canonical cytokine that induces macrophage M2 polarization. We also found that miR-382 knockdown with IL-4 treatment largely decreased CD206 expression in Raw264.7 cells (**Figure 6E**). In BMDMs, AA induced the increase in CD206 and Fizz1 expression in WT BMDMs, but it was inhibited in miR-382 KO BMDMs (**Figures 6F, G**). Of note, knockdown of miR-382 with AA treatment upregulated the MFI of CD86, compared with the anti-scramble +AA group (**Figure S5A**). However, we found no difference in the CD86 expression between WT and KO BMDMs after AA treatment (**Figure S5B**). Thus, in both *in vivo* and *in vitro* experiments, out data showed that miR-382 may exert little effect on macrophage M1 polarization but is important in macrophage M2 polarization in AAN.

In addition, KO BMDMs exhibited significantly decreased phosphorylation of STAT3 in Y705 and S727 after AA treatment, compared with WT BMDMs, while there was no difference in total STAT3 expression between WT and KO BMDMs in AA induction, indicating that miR-382 would regulate the phosphorylation of STAT3 instead of transcription or translation of STAT3 (**Figures 6A, H**). Accordingly, SIRP-α in macrophages was decreased in AA induction but was upregulated significantly after miR-382 knockdown, compared with the anti-scramble group (**Figures 6A, I**). However, the relationship among miR-382, SIRP-α, and STAT3 remained vague.

### 3.7 Overexpression of miR-382 Promotes Macrophage M2 Polarization *In Vitro*

To explore whether miR-382 alone could promote macrophage M2 polarization, miR-382 mimic was transfected into Raw264.7 cells and NC served as control. The expression of miR-382 elevated significantly after transfecting miR-382 mimic (**Figure 6K**). Overexpression of miR-382 alone upregulated phosphorylated STAT3 in Y705 and S727 but preserved total STAT3 expression, which was consistent with results above (**Figures 6J, L, M**). Additionally, overexpression of miR-382 suppressed the protein of SIRP-α in Raw264.7 cells (**Figures 6J, N**), indicating the reciprocal suppression relationship between miR-382 and SIRP-α. Moreover, overexpression of miR-382 promoted macrophage M2 polarization but exerted no effect on M1 polarization without AA induction (**Figure 6O**, **Figure S5C**). Overexpression of miR-382 could also upregulate the mRNA level of Arg-1, Fizz1, and Ym-1 in Raw264.7 cells (**Figure 6P**).

### 3.8 miR-382 Promotes Macrophage M2 Polarization Through Activation of STAT3 *via* Targeting SIRP-α

**Figure 7:**
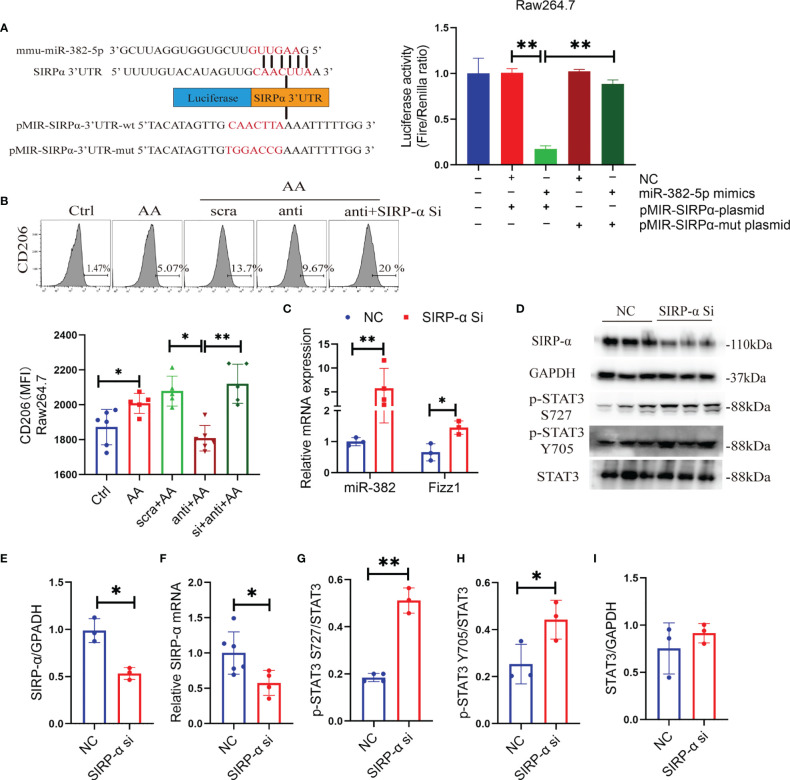
miR-382 promotes macrophage M2 polarization through activation of STAT3 *via* targeting SIRP-α. **(A)** Sequence within the SIRP-α 3′ UTR targeted by miR-382. Mutations were induced in the SIRP-α 3′UTR sequence. Luciferase activity was quantified in Raw264.7 cells of the control, NC+ p-MIR-SIRPα plasmid, miR-382 mimics+ p-MIR-SIRPα plasmid, NC+ p-MIR-SIRPα-mut plasmid, and miR-382 mimic+ p-MIR-SIRPα-mut plasmid groups. Dual-luciferase activity was measured using a Dual-Glo Luciferase Assay System; n = 3 per group. **(B)** Flow cytometry of CD206^+^ macrophages in Raw264.7 cells of the control, AA, AA+anti-scramble, AA+anti-miR-382, and AA+anti-miR-382+SIRP-α siRNA groups; MFI of CD206^+^ macrophages in Raw264.7 among these groups. **(C)** Transcript levels of miR-382 and Fizz1 in Raw264.7 with SIRP-α siRNA transfection. U6 was used as an endogenous control of miR-382. 18s served as standard of Fizz1. **(D)** Images of Western blot for SIRP-α, p-STAT3 Y705, p-STAT3 S727, and STAT3 in Raw264.7 cells in NC and SIRP-α siRNA groups. GAPDH served as standard. **(E, F)** Quantification of protein and mRNA levels for SIRP-α in Raw264.7 cells in NC and SIRP-α siRNA groups. GAPDH and 18s served as standard, respectively. **(G–I)** Quantification of protein expression for p-STAT3 S727, p-STAT3 Y705, and STAT3 in Raw264.7 cells in NC and SIRP-α siRNA groups. *P < 0.05; **P < 0.01; ANOVA.

Signal-regulatory protein alpha (SIRP-α) is a receptor expressed on macrophage, which could combine with CD47 and regulate the phagocytosis of macrophage (28). The protective role of SIRP-α was reported in both acute and chronic kidney diseases (29–31). Enhanced STAT3 phosphorylation was observed in tumor-exposed SIRP-α knockdown macrophage (32). It was predicted that SIRP-α is a target of miR-382 using TargetScan (http://www.targetscan.org/). Therefore, it was hypothesized that miR-382 activates STAT3 possibly by targeting SIRP-α. To prove the relationship between miR-382 and SIRP-α, a dual-luciferase reporter assay was performed in Raw264.7 cells. In comparison with the NC+ pMIR-SIRP-α plasmid group, luciferase activity was suppressed significantly in the miR-382-5p mimic+ pMIR-SIRP-α plasmid group, indicating that miR-382-5p could downregulate SIRP-α. As opposed to the miR-382-5p mimic+ pMIR-SIRP-α plasmid group, the downregulation of luciferase activity was hindered in the miR-382-5p mimic+ pMIR-SIRP-α-mut plasmid group, suggesting that miR-382-5p targets SIRP-α *via* binding to CAACUUA in the 3′UTR (**Figure 7A**).

Knockdown of miR-382 hindered AA-induced macrophage M2 polarization but was recovered after SIRP-α siRNA transfection (**Figure 7B**), demonstrating that miR-382 regulated the polarization of macrophage possibly *via* suppression of SIRP-α. In addition, knockdown SIRP-α alone significantly upregulated miR-382 expression and increased Fizz1 (**Figure 7C**), suggesting that downregulation of SIRP-α promotes macrophage M2 polarization and SIRP-α may have a positive feedback on miR-382.

To address whether SIRP-α plays a role on the activation of STAT3 signaling, SIRP-α expression in Raw264.7 cells was suppressed *via* siRNA transfection. Both mRNA and protein levels of SIRP-α were inhibited significantly *via* SIRP-α siRNA transfection (**Figures 7D–F**). Furthermore, SIRP-α knockdown also increased the ratio of p-STAT3 S727/STAT3 and p-STAT3 Y705/STAT3, while total STAT3 was preserved (**Figures 7D, G–I**). Collectively, miR-382 suppressed SIRP-α by combining its 3′UTR and downregulation of SIRP-α further promoted phosphorylation of STAT3 signaling.

### 3.9 miR-382 Indirectly Activates STAT3 and Gets Involved in AA-Induced CKD

**Figure 8:**
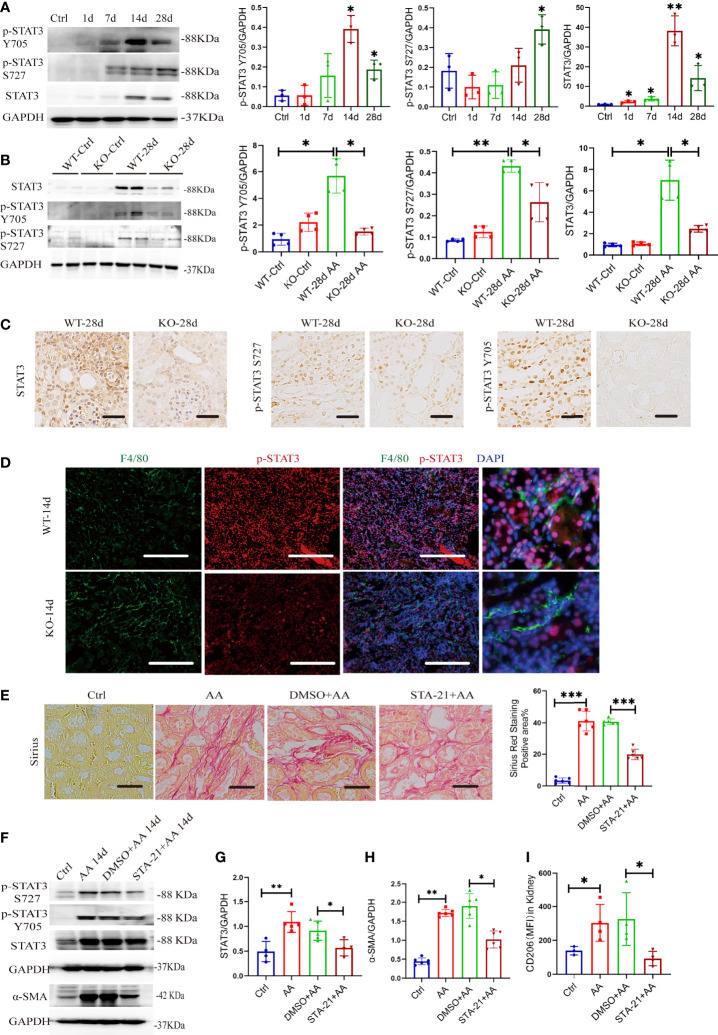
miR-382 indirectly activates STAT3 and gets involved in AA-induced CKD. **(A)** Images and quantification of Western blot for p-STAT3 Y705, p-STAT3 S727, and STAT3 in renal tissues in the time course of AAN. GAPDH served as standard. **(B)** Images and quantification of Western blot for p-STAT3 Y705, p-STAT3 S727, and STAT3 in renal tissues from WT and KO mice of 28 days AAN. GAPDH served as standard. **(C)** Immunohistochemistry staining with antibodies against p-STAT3 Y705, p-STAT3 S727, and STAT3 in renal sections between WT and KO mice of 28 days AAN. Scale bars, 200 μm. **(D)** Images of co-staining for F4/80, p-STAT3, and DAPI in renal sections from WT-14d and KO-14d. F4/80 was stained as green; p-STAT3 was stained as red. Nucleic was stained as blue. Scale bars, 100 μm. **(E)** Images of Sirius Red staining in these groups. Quantification of mean positive area for Sirius Red staining in these groups. Scale bars, 200 μm. **(F)** Images of Western blot for p-STAT3 Y705, p-STAT3 S727, STAT3, and α-SMA in kidney from control, AA 14 days, DMSO+ AA 14 days, and STA-21+ AA 14 days groups. GAPDH served as standard. **(G, H)** Quantification of western blot for STAT3 and α-SMA in control, AA 14 days, DMSO+ AA 14 days, and STA-21+ AA 14 days groups. GAPDH served as standard. **(I)** MFI of CD206+ macrophages in kidney from control, AA 14 days, DMSO+ AA 14 days and STA-21+ AA 14 days groups. *P < 0.05; **P < 0.01; ***P < 0.001; ANOVA.

Macrophage polarization is regulated by various transcriptional factors, such as STATs, PPARs, KLFs, and C/EBP β (8). Herein, STAT3 promotes M2-type macrophages in the progression of carcinoma (33–35). The phosphorylation of STAT3 at Y705 and S727 is necessary for the activation of STAT3 (36). We first examined phosphorylated STAT3 and total STAT3 in kidneys from the time course of AAN in mice. Both p-STAT3 Y705 and total STAT3 protein increased significantly after 14 and 28 days of AAN, and p-STAT3 S727 significantly increased at 28 days of AAN (**Figure 8A**). AA was administrated in WT and KO mice for 28 days separately, injected with saline as controls. We found that AA induced the activation of STAT3 signaling but was remarkably impaired in KO mice after 28 days of AAN (**Figure 8B**). Immunohistochemistry (IHC) staining for STAT3 signaling showed that total STAT3 is distributed in the cytoplasm and nucleus of renal cells and that p-STAT3 is mainly located in the nucleus. The expression of STAT3 signaling significantly decreased in KO mice after 28 days of AAN, compared with WT 28-day AAN (**Figure 8C**). More details of IHC for STAT3 signaling among the control group and 14 days of AAN and 28 days of AAN from WT and KO mice are displayed in **Figure S6A**. Moreover, co-staining for F4/80 and p-STAT3 in renal sections indicated that p-STAT3 expression in renal macrophages was inhibited in KO mice, compared with WT mice (**Figure 8D**). Thus, our data indicated that miR-382 would regulate the activation of STAT3 signaling in the AAN model.

To further explore the relationship of STAT3, macrophage M2 polarization, and kidney fibrosis, STA-21, an inhibitor of STAT3 (37),was administrated in the AAN model. The strategy for STA-21 injection is shown in **Figure S7A**. Sirius Red staining showed a significant decrease in collagen deposition in renal interstitial fibrosis after inhibition of STAT3 signaling (**Figure 8E**). At the protein level, both p-STAT3 and total STAT3 were downregulated in renal after administration of STA-21 in 14 days of AAN (**Figures 8F, G**). The protein expression of α-SMA decreased remarkably with STA-21 treatment in 14 days of AAN, as detected by Western blot assay (**Figures 8F, H**). Also, immunohistochemistry staining for α-SMA, collagen I, fibronectin, and vimentin showed relieved kidney fibrosis after STA-21 treatment (**Figure S7C, D**). In addition, we also evaluated the polarization of macrophage in the kidney after STA-21 treatment. Compared with DMSO +AA 14 days, macrophage M2 polarization was reduced remarkably while macrophage M1 polarization was preserved in STA-21 +AA 14 days (**Figure 8I**, **Figure S7B**). Therefore, activation of STAT3 signaling was involved in AA-induced macrophage M2 polarization as well as progression of kidney fibrosis.

### 3.10 STAT3 Regulates Activation of Cultured Macrophages, Which Is Partly Mediated by miR-382

**Figure 9:**
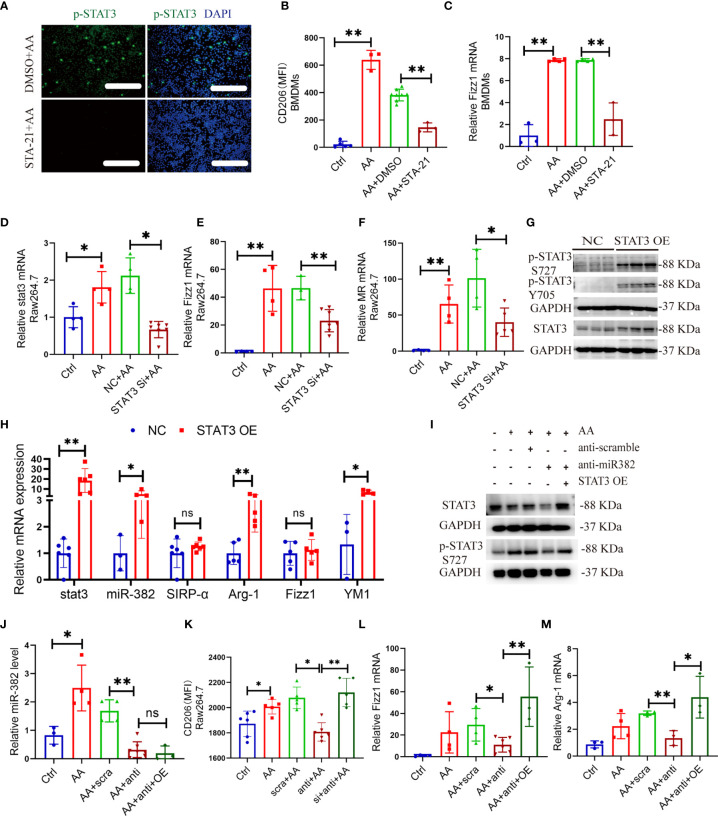
STAT3 regulates activation of macrophage *in vitro*, which is partly mediated by miR-382. **(A)** Images of immunofluorescence for p-STAT3 in DMSO+AA and STA-21+AA groups in Raw264.7 cells. Scale bars, 100 μm. **(B)** MFI of CD206+ macrophages in BMDMs from control, AA, DMSO+AA, and STA-21+AA groups. **(C)** Relative of mRNA expression of Fizz1 in BMDMs from control, AA, DMSO+AA, and STA-21+AA groups. **(D–F)** Relative of mRNA expression of stat3, Fizz1, and MR in Raw264.7 cells from control, AA, NC+AA, and STAT3 siRNA+ AA groups. **(G)** Representative immunoblot for p-STAT3 S727, p-STAT3 Y705, and STAT3 in Raw264.7 after overexpression of STAT3. GAPDH served as standard. **(H)** Relative of transcripts for stat3, miR-382, SIRP-α, Arg-1, Fizz1, and Ym-1 in Raw264.7 cells after STAT3 OE plasmid transfection, NC as control. **(I)** Images of Western blot for p-STAT3 and STAT3 in Raw264.7 cells from ctrl, AA, anti-scramble+ AA, anti-miR-382+ AA, and anti-miR-382+ STAT3 OE+ AA groups. GAPDH served as standard. **(J)** Abundance of miR-382 in ctrl, AA, anti-scramble+ AA, anti-miR-382+ AA, and anti-miR-382+ STAT3 OE+ AA groups. U6 served as standard. **(K)** MFI of CD206+ macrophages in ctrl, AA, anti-scramble+ AA, anti-miR-382+ AA, and anti-miR-382+ STAT3 OE+ AA groups. **(L, M)** Relative mRNA of Fizz1 and Arg-1 in ctrl, AA, anti-scramble+ AA, anti-miR-382+ AA, and anti-miR-382+ STAT3 OE+ AA groups. 18s served as standard. *P < 0.05; **P < 0.01; ANOVA.

Given that activation of STAT3 regulated AA-induced kidney fibrosis and renal macrophage M2 polarization, we further explored the roles of STAT3 in the activation of macrophage *in vitro*. The genetic knockdown or pharmacological inhibition of STAT3 was performed by STAT3 siRNA (NC as control) and STA-21 (DMSO as control) *in vitro*. The efficiency of STA-21 (STAT3 inhibitor) was identified *via* immunofluorescence for p-STAT3 in Raw264.7 between DMSO+AA and STA-21+AA groups (**Figure 9A**). In BMDMs, AA induced a significant expression of CD206 and Fizz1 but was inhibited by STA-21 (**Figures 9B, C**). In Raw264.7, AA increased the mRNA levels of stat3, Fizz1, and MR but they were suppressed by STAT3 siRNA (**Figures 9D–F**). The pharmacological inhibition of STAT3 in BMDMs also hindered macrophage M1 polarization by downregulating CD86 and iNOS (**Figure S8A, B**). Additionally, STAT3 was overexpressed by transfecting STAT3 OE plasmid in Raw264.7 cells. Both protein expression of p-STAT3 S727, p-STAT3 Y705, and STAT3 and mRNA level of *stat3* were upregulated significantly after STAT3 OE transfection (**Figures 9G, H** and **Figures S9A–C**). Overexpression of STAT3 alone could upregulate Arg-1 and Ym-1 as well as abundance of miR-382 but exerted no effect on the expression of SIRP-α (**Figure 9H**).

Moreover, the protein expression of p-STAT3 and STAT3 was inhibited after knockdown of miR-382 and was recovered after STAT3 OE (**Figure 9I**). Furthermore, the abundance of miR-382, MFI of CD206, and mRNA levels of Fizz1 and Arg-1 were suppressed after downregulation of miR-382 and was recovered after STAT3 OE except for miR-382 (**Figures 9J–M**), which suggests that miR-382 would be upstream of STAT3 in macrophages. Interestingly, the MFI of CD86 and mRNA level of iNOS were preserved after knockdown of miR-382 but were upregulated after STAT3 OE (**Figures S10A, B**).

### 3.11 Cocultured Experiments Reveal a Crosstalk Between Macrophages and Renal Tubular Cells

**Figure 10:**
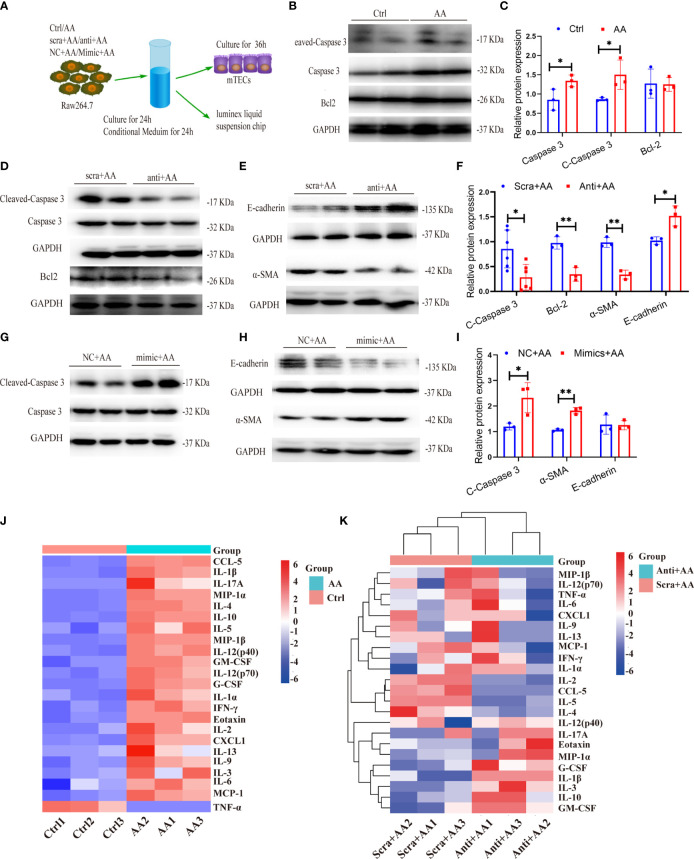
miR-382 in macrophage promotes the apoptosis and epithelial–mesenchymal transition (EMT) in kidney tubular cells. **(A)** Schematic diagram showing the experimental setup of performing coculture between Raw264.7 and MTECs. **(B, C)** Representative Western blot and quantification of Cleaved Caspase 3, Caspase 3, and Bcl-2 in MTECs with treatment of supernatant for 48 h from Raw264.7 (Ctrl or AA group). GAPDH served as standard. **(D–F)** Representative Western blot and quantification of Cleaved Caspase 3, Caspase 3, and Bcl-2 as well as E-cadherin and α-SMA in MTECs with treatment of supernatant for 48h from Raw264.7 (anti-scramble+ AA or anti-miR-382+ AA group). GAPDH served as standard. **(G–I)** Representative Western blot and quantification of Cleaved Caspase 3 and Caspase 3 as well as E-cadherin and α-SMA in MTECs with treatment of supernatant for 48 h from Raw264.7 (NC+ AA or Mimic+ AA group). GAPDH served as standard. **(J)** Luminex liquid suspension chip analysis of conditional medium (CM) collected from Raw264.7 in ctrl and AA groups. **(K)** Luminex liquid suspension chip analysis of conditional medium (CM) collected from Raw264.7 in anti-scramble+ AA and anti-miR-382+ AA groups. *P < 0.05; **P < 0.01; ANOVA.

As described above, miR-382 ablation significantly ameliorated kidney fibrosis and inhibited M2 polarization in renal tissue. Depletion of miR-382 in BMDMs resulted in lower M2 polarization than in wild-type BMDMs following AA exposure. As previously reported, the apoptosis and epithelial to mesenchymal transition (EMT) of tubular epithelia (38)would drive kidney fibrosis. Therefore, we hypothesized that depletion of miR-382 in macrophages may also protect tubular cells from apoptosis and EMT. Therefore, we cocultured macrophages with tubular epithelial fibrosis (**Figure 10A**). In the coculture system, we detected apoptosis-related proteins, such as cleaved caspase 3, total caspase 3, and Bcl-2, as well as EMT-related marker proteins, such as epithelial cell marker E-cadherin and mesenchymal marker α-SMA. The conditional medium (CM) from AA-treated macrophages upregulated cleaved caspase 3 and total caspase 3 protein expression in MTECs (**Figures 10B, C**). MiR-382 knockdown in macrophages downregulated the protein expression of cleaved caspase 3 and Bcl-2 in MTECs (**Figures 10D, F**). Knockdown of miR-382 in macrophages resulted in the preservation of the epithelial marker E-cadherin and the suppression of the mesenchymal marker α-SMA in MTECs (**Figures 10E, F**). In turn, overexpression of miR-382 in macrophages upregulated cleaved caspase-3 (**Figures 10G, I**). Overexpression of miR-382 in macrophages induced a decrease in E-cadherin and promoted α-SMA expression (**Figure 10H, I**). Therefore, these results indicated that upregulation of miR-382 in macrophages would promote apoptosis and EMT of renal tubular epithelial cells.

To further explore the potential regulators by which AA-stimulated macrophages promoted injuries of tubular epithelial cells, Luminex liquid suspension chip detection was applied to compare the differential expression of 23 common cytokines or chemokines in conditional medium (CM) from Raw264.7 in ctrl, AA, anti-scramble+ AA, and anti-miR-382+AA groups. CCL-5, IL-1β, IL-17A, MIP-1α, IL-4, and IL-10 were the six most highly upregulated proteins in AA-CM, compared with Ctrl-CM (**Figure 10J**). IL-2, CCL-5, IL-5, and IL-4 were downregulated notably in anti-miR-382+AA-CM, compared with anti-scramble+ AA-CM. IL-1β and G-CSF were upregulated significantly in anti-miR-382+AA-CM, compared with anti-scramble+ AA-CM (**Figure 10K**). As reported in recent literature, CCL-5 and IL-4 were modulated by NGAL produced from macrophages and were proved to play a critical role in renal fibrosis (39). As our results show, CCL5 and IL-4 were elevated in AA-CM (Ctrl-CM as control) but were suppressed in anti-miR-382+AA-CM (anti-scramble+ AA-CM as control), suggesting that CCL-5 and IL-4 would be the critical mediators in the involvement of miR-382 on AA-induced CKD. More specific evidence will be learned in our following study.

## 4 Discussion

While the nephrotoxic and carcinogenic effects of AA have been recognized for many years, the exact mechanism through which AA participates in CKD remains largely unknown (40). We recently demonstrated that the upregulation of miR-382 promoted the EMT in tubular epithelial fibrosis and further exacerbated interstitial fibrosis in AAN *via* the PTEN/AKT pathway (11). In the present study, we found upregulation of miR-382 in both renal macrophages after AAN and cultured macrophages with AA treatment. Knockdown of miR-382 in Raw264.7 or knockout of miR-382 in BMDMs partly inhibited AA-induced M2-like macrophages while overexpressed miR-382 could upregulate the expression of M2-related genes. *In vivo*, systemic knockout of miR-382 significantly alleviated AA-induced CKD, accompanied by decreased M2 phenotype macrophages. Subsequently, macrophage-specific knockout of miR-382 mice exhibited less M2-like macrophage and α-SMA expression in renal after 14 days of AAN. Further, coculture of macrophages and MTECs revealed that upregulation of miR-382 in the former triggered apoptosis and the EMT in the latter cell type. Biochip detection suggested that IL-4 and CCL-5 would be involved in the contribution of miR-382 to AA-induced CKD. Our findings highlight a more pathophysiological molecular mechanism for CKD and may facilitate the identification of promising therapeutic targets for the treatment of nephrotoxicity-related CKD.

The significance of macrophage polarization in CKD has been previously explored. Our experiments show that during the early stage of AA induction, the number of macrophages in renal tissue increased significantly, yet their M0 phenotype was preserved. In the chronic phase of AAN, classically activated M1 and alternatively activated M2 coexisted in fibrotic kidneys. Accordingly, M1 served as pro-inflammatory and M2 as anti-inflammatory and profibrotic (41). It was reported that F4/80^+^CD11b^+^CD206^+^ M2 macrophages promoted the excessive accumulation of the extracellular matrix and interstitial fibrosis *via* TGF-β1/Smad2/3 signaling (42). Conversely, it has been reported that M1 macrophages exhibited strong therapeutic effects in the amelioration of liver fibrosis (43). Our cell-based experiment also revealed that the knockdown of miR-382 upregulated CD86 expression in Raw264.7 cells, thus proposing another explanation for the role of miR-382 in renal fibrosis.

Concerning the role of miR-382 in AA-induced macrophage M2 polarization, by subjecting the miR-382 knockout mice as well as macrophage-specific miR-382 depletion mice (**Figure S2**) in the AAN model, we found that M2-like macrophages were significantly inhibited and AA-induced renal fibrosis was alleviated. Adoptive transfer miR-382 OE macrophages in mice triggered macrophage M1 to M2 transition and augmented AA-induced CKD. In addition, *via* biochip detection for supernatant of macrophage (**Figure 10K**), several cytokines or chemokines would be modulated by miR-382 in AA stimulation, such as CCL-5, IL-4, IL-13, IL-5, and IL-2. Interestingly, IL-4 and IL-13 are classical cytokines which have been proved to regulate macrophage M2 polarization (8, 44). As reported in recent literature, CCL-5 and IL-4 were modulated by NGAL produced from macrophages and were proved to play a critical role in renal fibrosis (39). In breast cancer, IL-2 produced by myofibroblasts would promote post-radiation fibrosis (45). IL-5 was reported as a promoter in both liver and pulmonary fibrosis (46, 47). Therefore, even though the decreases of CD206 MFI were not impressive enough in miR-382 knockdown Raw264.7 and miR-382 knockout BMDMs, the subsequent effects were still notable. In addition, miR-382 seemed to be more responsible for IL-4-induced M2 polarization (**Figure 6E**) than that of AA. As previously reported, serum IL-4 levels are associated with disease severity in patients with membranous nephropathy (48). However, whether IL-4 is involved in AA nephropathy still remained unknown. More efforts would be made to explore the role of miR-382 and IL-4 in AAN.

Depletion of miR-382 in mice significantly reserved AA-induced kidney fibrosis and inhibited the M2 phenotype while preserving the M1 phenotype. Therefore, we proposed that M2 macrophages play a prominent role in the progression of kidney fibrosis in AAN, with miR-382 being involved in AAN possibly *via* promoting the M2 polarization of macrophages, in accordance with our present findings.

MicroRNAs are endogenous, small non-coding RNAs with critical roles in kidney disease (49, 50); however, these studies focused primarily on miRNA functions in fibroblasts. Nevertheless, macrophages but not fibroblasts are a major cell type that secretes profibrotic cytokines such as TGF-β1. Therefore, modulating the polarization of macrophages may be more effective for preventing tissue fibrosis. Several studies have revealed the involvement of microRNAs in macrophage polarization during kidney disease. For example, exosomal miRNA-19b-3p from tubular epithelial fibrosis promotes M1 macrophage activation in kidney injury (51). The microRNA miR-16 was reported to induce the M1 differentiation of mouse peritoneal macrophages (52). In addition, macrophage-specific lncRNAs MAARS and MM2P were reported to act as important regulators of macrophage polarization or apoptosis (53, 54). In our study, administration of anti-miR-382 oligo significantly suppressed M2 polarization of macrophages. This finding may have clinical applications for the therapy of patients with CKD.

Several transcription factors have been reported to promote M2 polarization, including IRF4, C/EBP-β, KLF4, STAT3, STAT6, and the PPARγ receptor, among others (55). Our data explicitly demonstrated that miR-382 depletion suppressed AA-induced STAT3 activation *in vivo* and *in vitro*. The signal transducer and activator of transcription 3 (STAT3) protein, a member of the STAT family of transcription factors, translocates to the nucleus following phosphorylation and is involved in several pathological processes (56). STAT3 promotes tumor growth, invasion, and tumor-associated macrophage proliferation (37, 57). Apart from miR-382, STAT3 was also regulated by miR-1246, IL-6, IL-10, nuclear factor kappa-B p65, or ERK and promoted M2-type macrophages in the progression of carcinoma (33–35). The association of STAT3 and fibrosis has been reported in STZ-induced kidney fibrosis and liver fibrosis (58, 59). A high level of phosphorylated STAT3 was found in fibrotic peritoneal fibrosis from patients with long-term peritoneal dialysis (PD) (60). In our study, the *in vivo* inhibition of STAT3 suppressed the M2 phenotype of macrophages in AA-induced kidneys. Overexpression of STAT3 *in vitro* significantly increased Arg-1 and Ym-1. However, Fizz1 was preserved after STAT3 OE while it was upregulated significantly after STAT3 OE in AA induction, suggesting that STAT3 merely upregulated the expression of Fizz1 in the AA stimulation. It is our hypothesis that regulation of Fizz1 was coactivated by STAT3 and other signaling pathways such as STAT6 (61). Yet no evidence of direct regulation of STAT3 by miR-382 has been reported. However, we further observed SIRP-α, a target of miR-382, to be a critical mediator in the phosphorylation of STAT3 at Y705 and S727 in macrophages (32).

Signal regulatory protein α (SIRPα) is a cell-surface protein mainly expressed on myeloid cells, including macrophage and dendritic cells (62). SIRPα binds to CD47, a receptor frequently overexpressed on cancer cells, and this interaction provides a “do-not-eat-me” signal to prevent phagocytosis, which plays a pivotal role in tumor progression (28). However, the roles of SIRPα on macrophage polarization are obscure. Our study shows SIRP-α to be fully expressed on macrophages, with a significant decrease in expression observed following AA treatment. SIRP-α knockdown was seen to polarize macrophages to the M2 phenotype. Additionally, the protective role of SIRP-α was reported in both acute and chronic kidney diseases as well as cardiac hypertrophy and fibrosis (29–31). Therefore, macrophage SIRP-a may be a promising target for AA-induced CKD therapy, which could be confirmed in future studies. Our results taken together indicated that miR-382/SIRP-α/STAT3 may represent a major signaling axis in the process of AA-induced M2 macrophage polarization. We intend to conduct future studies on the selective depletion of miR-382 in macrophages of mice to confirm our observations from the cell-based experiments in the present study.

However, the limitation of our research is the use of male mice. More work should be contributed to show differences between females and males. Although macrophage depletion experiments were performed to support the essential role of macrophage on AA-induced CKD, more specific evidence should be provided about the role of M2-like macrophage on AAN. Through the biochip sequencing of cytokines and chemokines, we found the critical mediators in AAN but more intervention of IL-4 and CCL-5 has to be conducted in the future. Moreover, interaction among miR-382, SIRP-α, and macrophage M2 polarization could be further learned.

**Figure 11:**
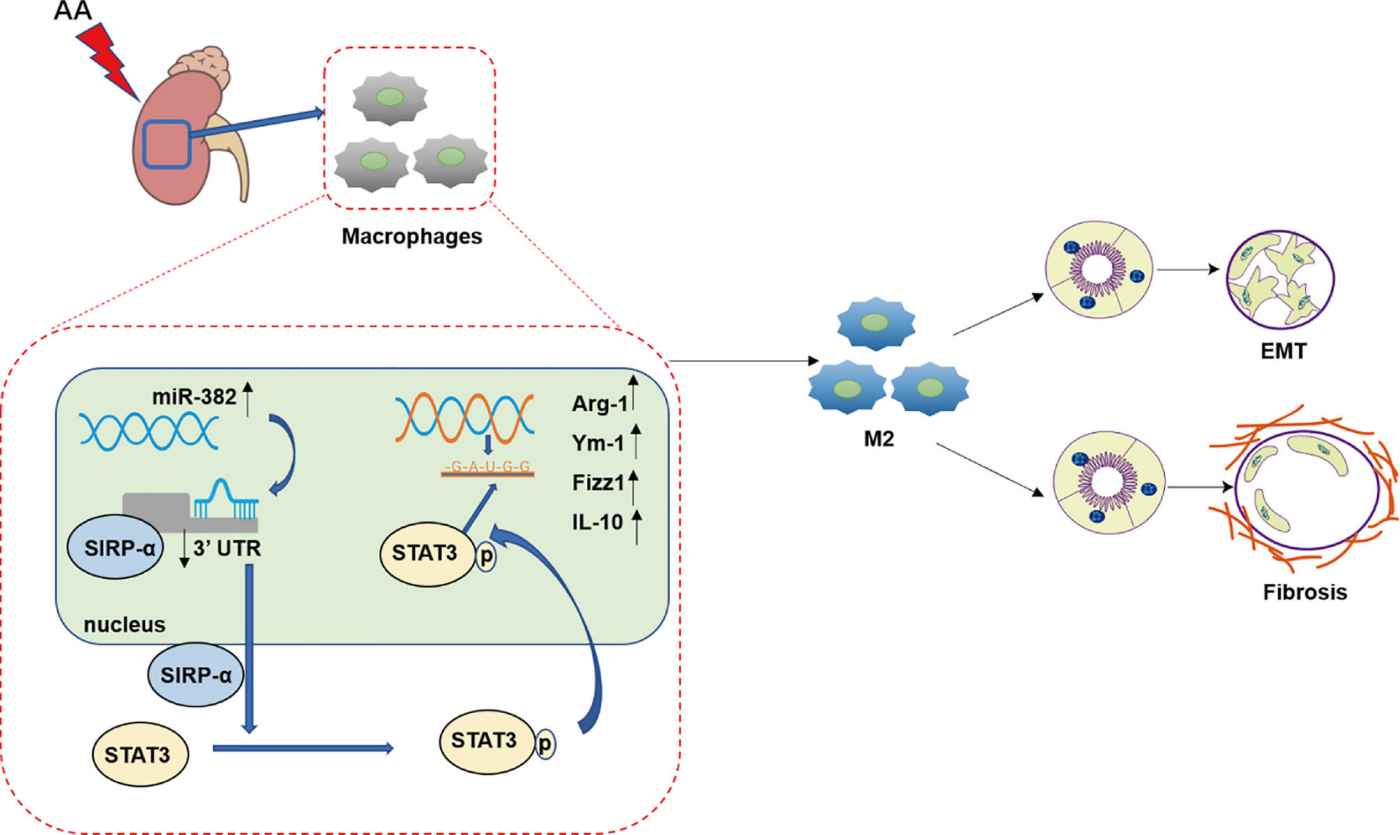
Working model of this study. In the progression of aristolochic acid nephropathy, the expression of miR-382 in kidney macrophage increased significantly and then suppressed signal regulatory protein α (SIRP-α) by combining 3′UTR of SIRP-α. Then, downregulation of SIRP-α activated the signal transducer and activator of transcription 3 (STAT3) by promoting the phosphorylation of STAT3 at S727 and Y705. Phosphorylated STAT3 translocated into nucleic and acted as a transcription factor, which was responsible for M2-like macrophage activation. M2-like macrophage was involved in epithelial to mesenchymal transition (EMT) of renal tubular epithelial as well as pro-fibrotic effects in aristolochic acid-induced kidney fibrosis.

In conclusion, the present study proposes a promising mechanism of AAN, wherein the upregulation of miR-382 induced by AA promotes alternative macrophage activation and subsequent interstitial fibrosis through enhanced SIRP-α-mediated STAT3 phosphorylation (**Figure 11**). Targeting macrophage miR-382 may provide a useful strategy for the attenuation of kidney tubulointerstitial fibrosis.

## Data Availability Statement

The original contributions presented in the study are included in the article/**Supplementary Material**. Further inquiries can be directed to the corresponding authors.

## Ethics Statement

The animal study was reviewed and approved by the Institutional Animal Care and Use Committee of Fudan University.

## Author Contributions

XW contributed to the conception and design, performance of the experiments, acquisition of data, or analysis and interpretation of data. She also drafted the article or revised it critically for important intellectual content. PJ, YF, and XD contributed to the conception and design, revised the article, and made the final approval of the version to be published. TR, ZZ and SX assisted in performing the experiments, acquisition of data, or analysis and interpretation of data and made the final approval of the version to be published. YZ assisted in drawing the working model. YS assisted in analysis and interpretation of data. SB and YL assisted in performing the experiments and final approval of the version to be published. All authors contributed to the article and approved the submitted version.

## Funding

This work was supported by the Science and Technology Commission of Shanghai (14DZ2260200) and the National Natural Science Foundation of China grants 91849123 (to XD) and 81870466 (to PJ).

## Conflict of Interest

The authors declare that the research was conducted in the absence of any commercial or financial relationships that could be construed as a potential conflict of interest.

## Publisher’s Note

All claims expressed in this article are solely those of the authors and do not necessarily represent those of their affiliated organizations, or those of the publisher, the editors and the reviewers. Any product that may be evaluated in this article, or claim that may be made by its manufacturer, is not guaranteed or endorsed by the publisher.

## Abbreviations

AAN, aristolochic acid nephropathy; miR-382, microRNA-382; BMDMs, bone marrow-derived macrophage; SIRP-α, signal regulatory protein α; STAT3, signal transducer and activator of transcription 3; CKD, chronic kidney disease; EMT, epithelial–mesenchymal transition; TGFβ1, transforming growth factor β1; LC, liposomal clodronate; MTECs, mouse renal tubular epithelial cells; IL-10, interleukin-10; IL-6, interleukin-6; TNF-α, tumor necrosis factor-α.
