# Supplementary material for: MicroRNA-382 Promotes M2-Like Macrophage via the SIRP-α/STAT3 Signaling Pathway in Aristolochic Acid-Induced Renal Fibrosis
Source: Front Immunol. 2022 May 2;13:864984. doi: 10.3389/fimmu.2022.864984 (PMC9108237; doi:10.3389/fimmu.2022.864984)
Supplement: Supplementary file 1 [file DataSheet_1.pdf]

Supplementary Table S1. Primer sequences used for real-time PCR analysis

Gene symbol	Sequence
Fizz1	Sense: 5’- ATGACTGCTACTGGGTGTGC-3’,<br>Antisense: 5’- TAAGCACAGGCAGTTGCAAG-3’
Arg-1	Sense: 5’- CTCCAAGCCAAAGTCCTTAGAG -3’,<br>Antisense: 5’- AGGAGCTGTCATTAGGGACATC -3’
Ym-1	Sense: 5’- TGAACGTACAGCTGGGATCTTC -3’,<br>Antisense: 5’- TTCTGCATTCCAGCAAAGGC -3’
IL-10	Sense: 5’- GCTCTTACTGACTGGCATGAG -3’,<br>Antisense: 5’- CGCAGCTCTAGGAGCATGTG -3’
IL-6	Sense: 5’-TAGTCCTTCCTACCCCAATTTCC -3’,<br>Antisense: 5’-TTGGTCCTTAGCCACTCCTTC -3’
TNF-α	Sense: 5’- CATGAGCACAGAAAGCATGATCCG -3’,<br>Antisense:5’-AAGCAGGAATGAGAAGAGGCTGAG -3’
iNOS	Sense: 5’- CAGATCGAGCCCTGGAAGAC -3’,<br>Antisense: 5’- CTGGTCCATGCAGACAACCT -3’
Col 1	Sense: 5’- GCTCCTCTTAGGGGCCACT-3’,<br>Antisense: 5’-CCACGTCTCACCATTGGGG -3’
TGF-β1	Sense: 5’- TGCTCGCTTTGTACAACAGC-3’,<br>Antisense: 5’-TCATAGATGGCGTTGTTGCG -3’
18s	Sense: 5’- CGGCTACCACATCCAAGGAA -3’,<br>Antisense:5’-CCTGTATTGTTATTTTTCGTCACTACCT -3’
MR	Sense: 5’-TTGCGTGCCATCGTTAAGAG -3’,<br>Antisense:5’-AACTGCCAAAGCTAGCTGTG-3’

STAT3	Sense: 5’-CAATACCATTGACCTGCCGAT -3’,<br>Antisense:5’-GAGCGACTCAAACTGCCCT-3’
SIRP-α	Sense: 5’-CCACGGGGAAGGAACTGAAG -3’,<br>Antisense:5’-ACGTATTCTCCTGCGAAACTGTA-3’

Supplementary Table S2. Primer sequences used for identification of transgenic mice

**Gene symbol**	**Sequence**
MiR-382	Sense: 5’- CCCCACCTCACTAACACTC-3’,<br>Antisense: 5’- ACATCCATACTTGGCTTCTC-3’
MiR-382 flox	Sense: 5’- AGTGTGAACTCTAGAACCAAGGAA -3’,<br>Antisense: 5’- CAACTTCTCTTCAAGTACCACAG -3’
Lyz Cre	P1: 5’- CCCAGAAATGCCAGATTA CG -3’,<br>P2: 5’- CTTGGGCTGCCAGAATTTCTC -3’<br>P3: 5’- TTACAGTCGGCCAGGCTGAC -3’

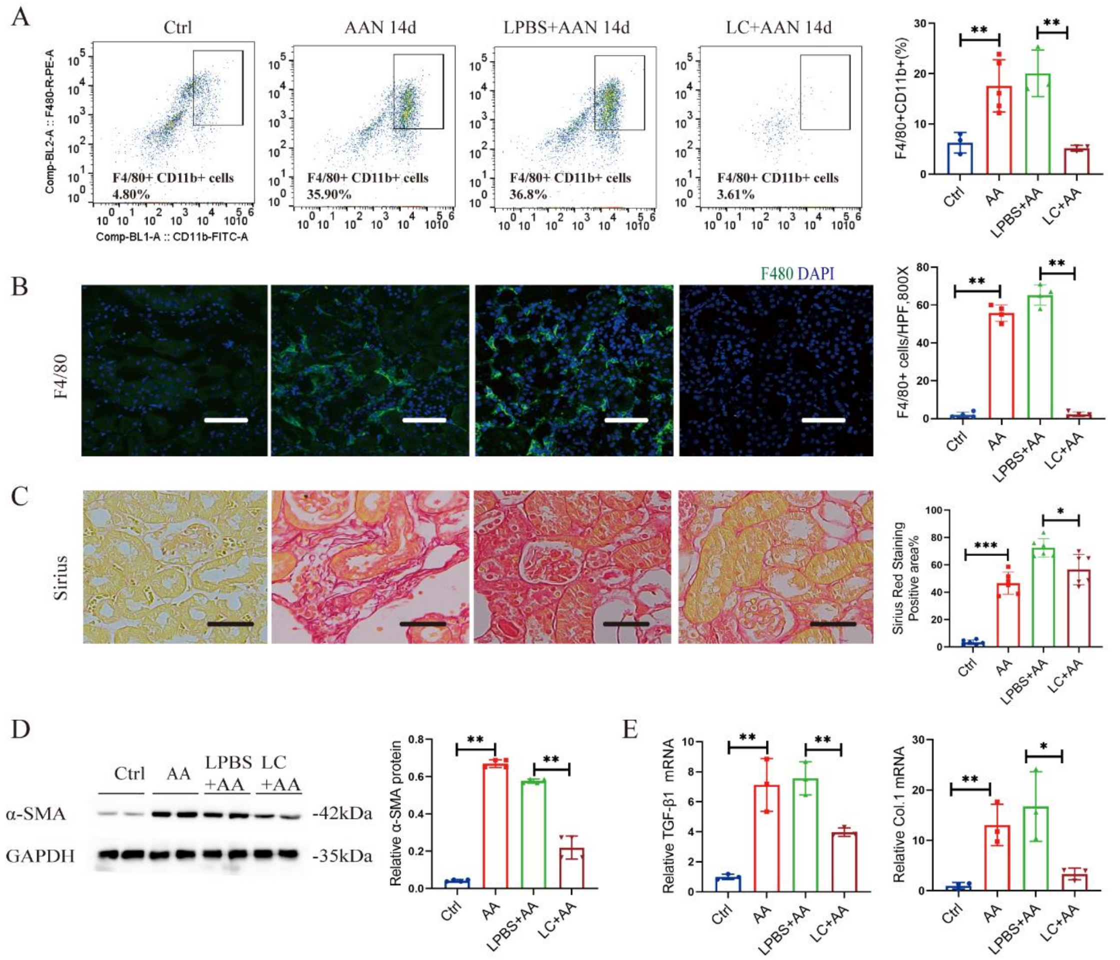

Supplementary Figure.1 Depletion of macrophages in AA nephropathy (A) Representative flow cytometry plots and percentage of CD11b+F4/80+ macrophages in control, AAN 14 days, LPBS+AAN 14 days and LC+AAN 14 days. (B) Immunofluorescence staining of anti-F4/80 of renal sections from normal group, AAN 14days group, LPBS +AAN group and LC+AAN group. Scale bars, 50μm. And F4/80 positive cells among groups were counted. (C) Representative images of western blot of α-SMA in renal in control, AAN 14 days, LPBS+AAN 14 days and LC+AAN 14 days. GAPDH served as standard. (D) Relative mRNA level of TGF-β1 and Collagen I in renal. 18s served as standard. * $P < 0.05$; ** $P < 0.01$; *** $P < 0.001$; ANOVA.

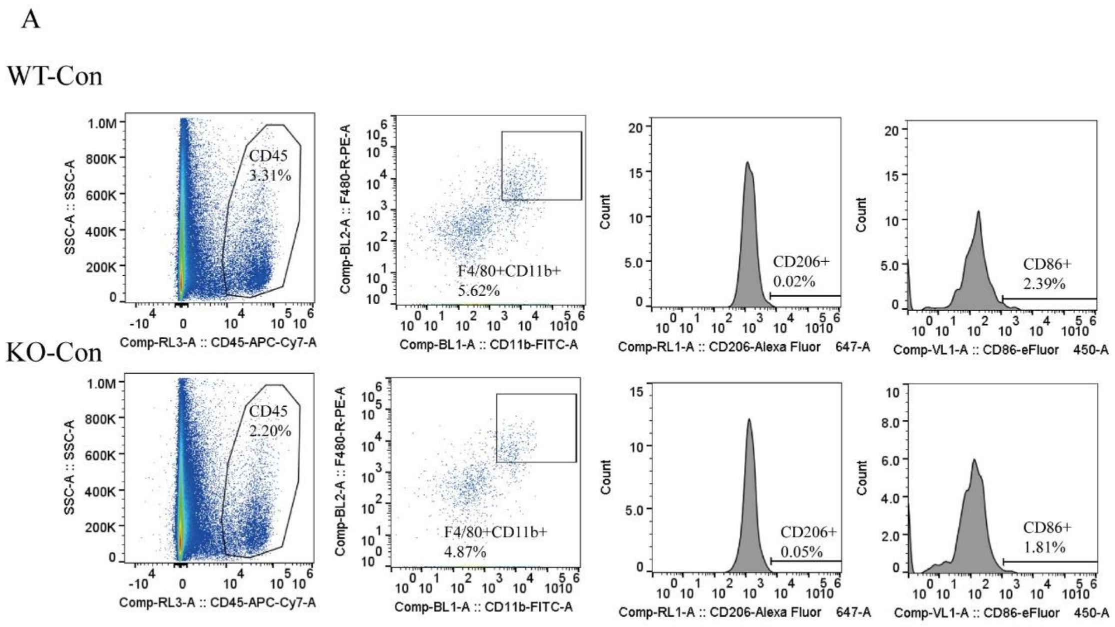

Supplementary Figure. 2 Representative flow cytometry images of normal kidneys from WT and KO mice. (A) Representative flow cytometry images for CD45+ leukocytes, CD11b+F4/80+ macrophages, CD86+ macrophages and CD206+ macrophages of normal kidneys from WT and KO mice.

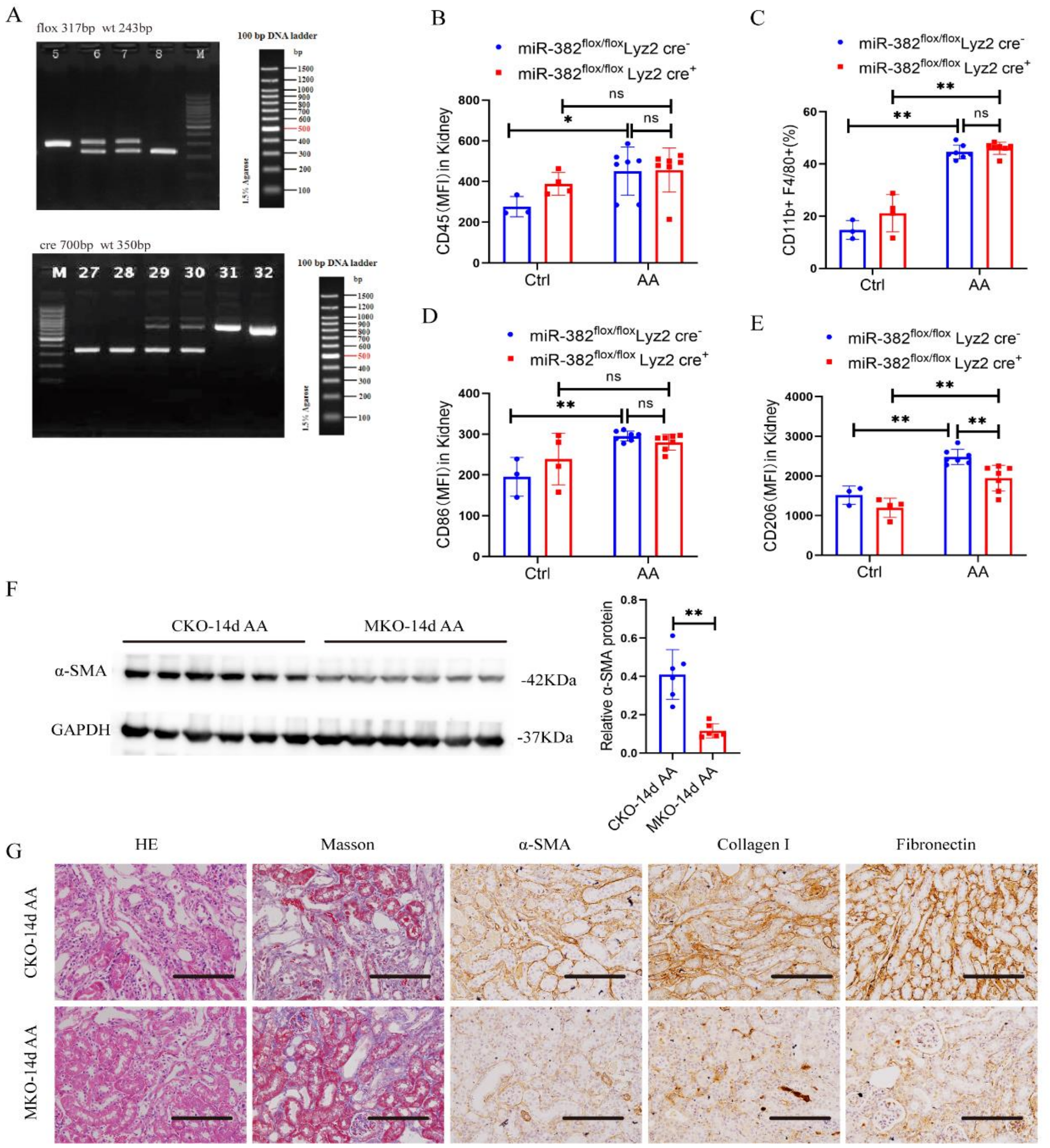

Supplementary Figure. 3 Macrophage-specific miR-382 depletion inhibits M2-like macrophages and alleviates AA-induced renal fibrosis. (A) Identification of miR-382$^{flox/flox}$Lyz2 Cre^{-} (CKO) and miR-382$^{flox/flox}$Lyz2 Cre^{+} (MKO) mice. Sample 5 was miR-382 flox homozygous (317bp). Sample 6 and 7 were miR-382 flox heterozygote (317bp/243bp). Sample 8 was wildtype mice (243bp). Sample 27 and 28 were wildtype mice (350bp). Sample 29 and 30 were Lyz2 Cre heterozygote (700bp/350bp). And sample 31 was Lyz2 Cre homozygous (700bp). (B) MFI of CD45+ leukocytes in renal among CKO-Ctrl, MKO-Ctrl, CKO-14d AAN and MKO-14d AAN groups. (C) Percentage of CD11b+F4/80+ macrophages in renal among CKO-Ctrl, MKO-Ctrl, CKO-14d AAN and MKO-14d AAN groups. (D) MFI of CD86+ M1-like macrophages in renal from CKO-Ctrl, MKO-Ctrl, CKO-14d AAN and MKO-14d AAN groups. (E) MFI of CD206+ M1-like macrophages in renal from CKO-Ctrl, MKO-Ctrl, CKO-14d AAN and MKO-14d AAN groups. (F) Western blot images and relative protein level of α-SMA in renal tissue between CKO and MKO mice after AA injection for 14 days. (G) Representative images of HE staining, Masson staining and IHC for α-SMA,

Collagen I and Fibronectin in renal between CKO-14d AAN and MKO-14d AAN. GAPDH served as standard. n=6 mice each group. ns $P >= 0.05$; * $P < 0.05$; ** $P < 0.01$; ANOVA.

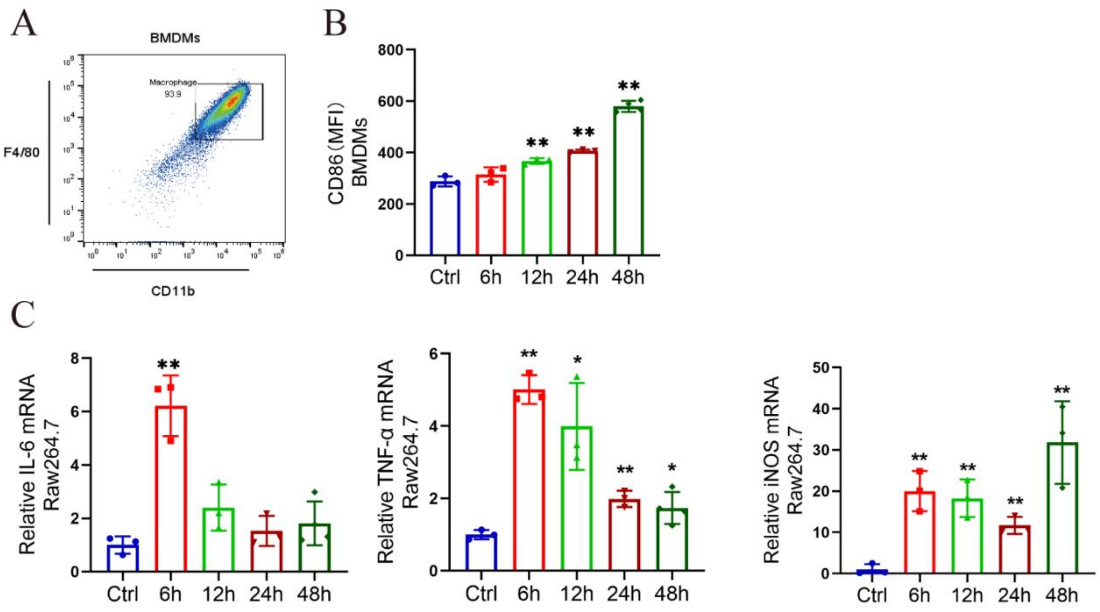

Supplementary Figure. 4 Identification of BMDMs; AA induces macrophage M1 polarization. (A) Identification of BMDMs by flow cytometry. **(B)** MFI of CD86+ BMDMs in the time course of AA treatment. **(C)** Relative mRNA levels of IL-6, TNF-α and iNOS in Raw264.7 cells in the time course of AA treatment. * $P < 0.05$; ** $P < 0.01$; ANOVA.

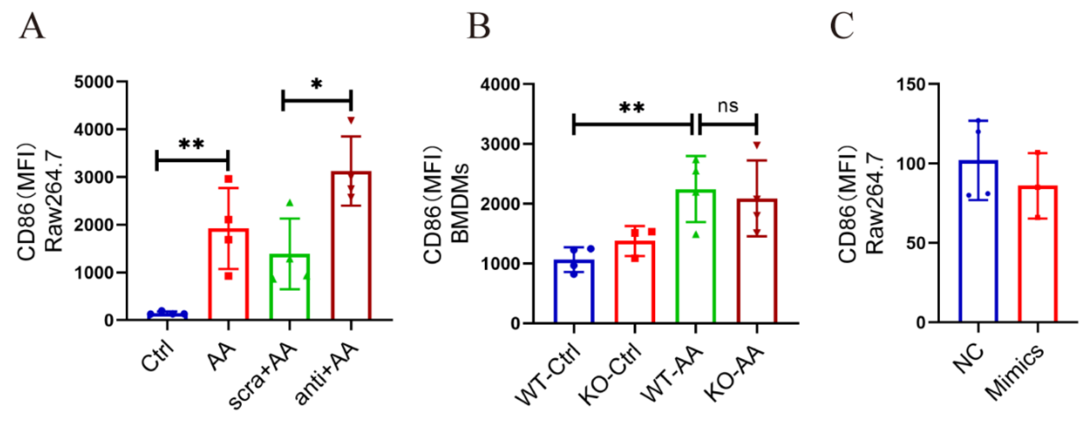

Supplementary Figure. 5 The role of miR-382 in macrophage M1 polarization (A) MFI of CD86+ M1-like macrophages in Raw264.7 among ctrl, AA, anti-scramble+ AA and anti-miR-382+ AA groups. **(B)** MFI of CD86+ macrophages in BMDMs among wt-ctrl, ko-ctrl, wt-AA and ko-AA groups. **(C)** MFI of CD86+ macrophages in Raw264.7 between NC and Mimics groups. ns $P >= 0.05$; * $P < 0.05$; ** $P < 0.01$; ANOVA.

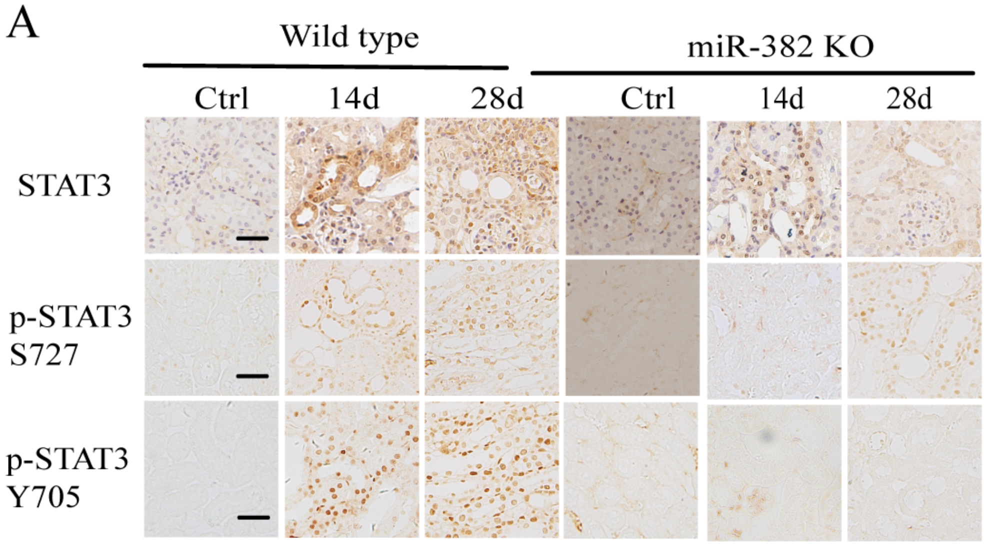

Supplementary Figure. 6 Suppression of miR-382 impaired activation of STAT3. **(A)** Images of immunohistochemical staining with antibodies against STAT3, p-STAT3 S727, and p-STAT3 Y705 in renal sections of WT and KO mice after AA injection for 14 and 28 days (NS injection as control). Scale bars, 20μm.

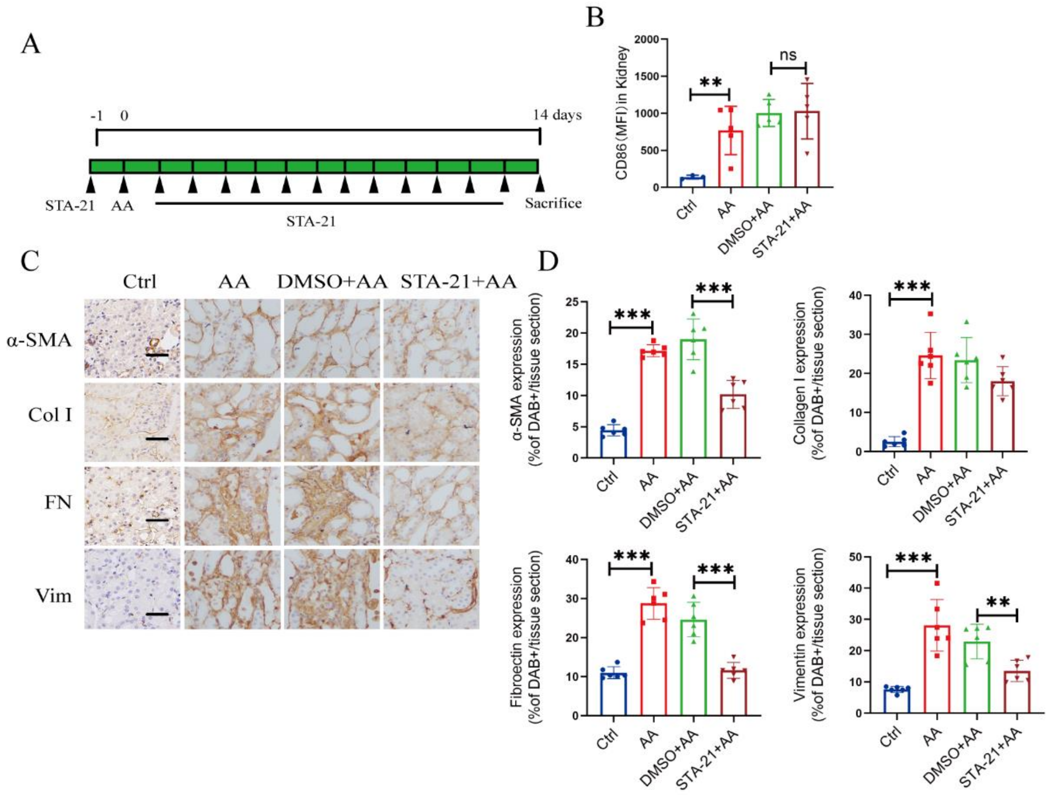

Supplementary Figure. 7 Strategy of STA-21 treatment in AAN mouse; Representative images of IHC for fibrosis protein following STA-21 treatment in AAN (A) Strategy for STA-21 administration in mice. STA-21 was injected intraperitoneally at a dose of 0.5 mg/kg the day before AA injection, and the same dose of STA-21 was administered once daily for 2 weeks. **(B)** MFI of CD86+ macrophages in renal tissue after STA-21 treatment determined via flow cytometry. **(C)** Representative images of IHC for vimentin, α-SMA, fibronectin, and collagen I staining in renal sections among ctrl, AA 14 days, DMSO+AA 14 days and STA-21+AA 14 days groups. **(D)** Quantification of positive area for vimentin, α-SMA, fibronectin, and collagen I staining in renal sections. ns $P >= 0.05$; ** $P < 0.01$; ***$P < 0.001$; ANOVA.

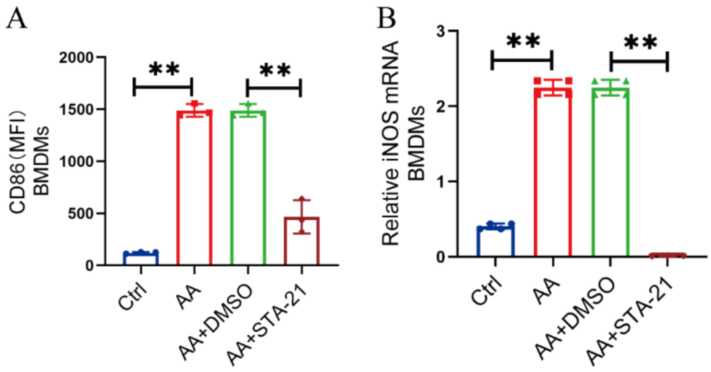

Supplementary Figure. 8 Pharmacological inhibition of STAT3 in BMDMs suppressed M1-like macrophages (A) MFI of CD86 in BMDMs from ctrl, AA, DMSO+ AA and STA-21+AA groups. **(B)** Relative mRNA level of iNOS in BMDMs from ctrl, AA, DMSO+ AA and STA-21+AA groups. ** $P < 0.01$; ANOVA.

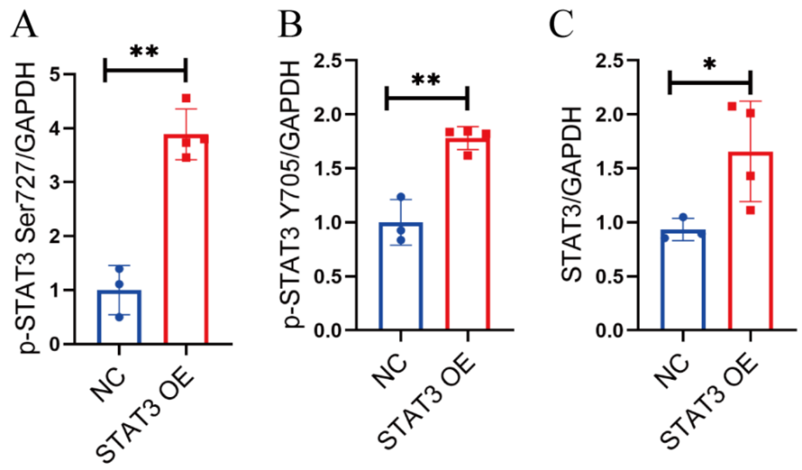

Supplementary Figure. 9 Quantification of protein expression of STAT3 signaling after STAT3 OE plasmid transfection. (A-C) Relative of p-STAT3 S727, p-STAT3 Y705 and STAT3 between NC and STAT3 OE groups in Raw264.7 cells. GAPDH served as standard. * $P < 0.05$; ** $P < 0.01$; ANOVA.

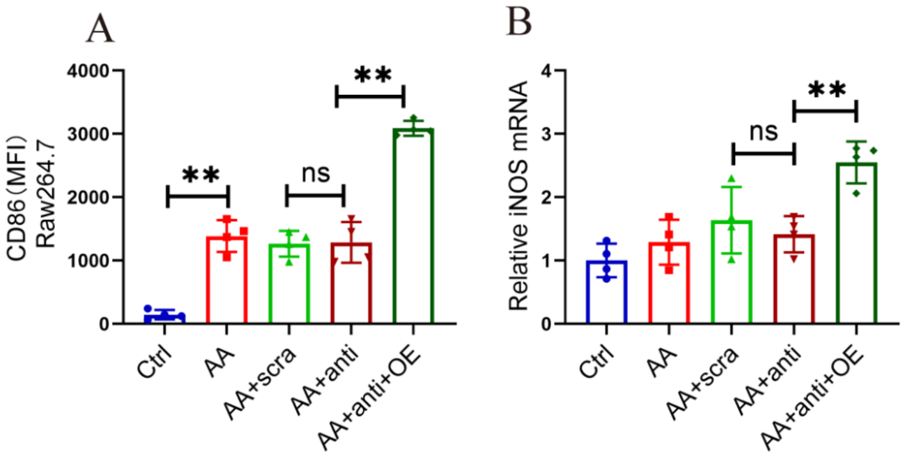

Supplementary Figure. 10 Macrophage M1 polarization after STAT3 overexpression. (A) MFI of CD86 in Raw264.7 from ctrl, AA, anti-scramble+ AA, anti-miR-382+ AA and STAT3 OE+ anti-miR-382+ AA groups. **(B)** Relative mRNA level of iNOS in Raw264.7 from ctrl, AA, anti-scramble+ AA, anti-miR-382+ AA and STAT3 OE+ anti-miR-382+ AA groups. ns P >=0.05; ** P < 0.01; ANOVA.

A

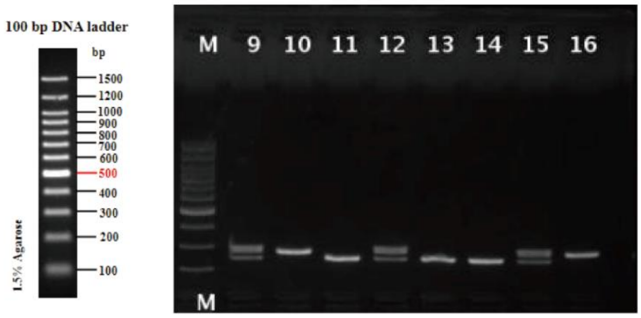

HE: 9, 12, 15
WT: 10, 16
KO: 11, 13, 14

Supplementary Figure. 11 (A) Identification of miR-382 knockout mice.
